# P-tau and neurodegeneration mediate the effect of β-amyloid on cognition in non-demented elders

**DOI:** 10.1186/s13195-021-00943-z

**Published:** 2021-12-15

**Authors:** Ling-Zhi Ma, Hao Hu, Zuo-Teng Wang, Ya-Nan Ou, Qiang Dong, Lan Tan, Jin-Tai Yu

**Affiliations:** 1 Department of Neurology, Qingdao Municipal Hospital, Qingdao University, Qingdao, 266071 China; 2 Department of Neurology and Institute of Neurology, Huashan Hospital, State Key Laboratory of Medical Neurobiology and MOE Frontiers Center for Brain Science, Shanghai Medical College, Fudan University, 12th Wulumuqi Zhong Road, Shanghai, 200040 China

**Keywords:** Alzheimer’s disease, β-amyloid, Biomarkers, Cognition

## Abstract

**Background:**

There are many pathological changes in the brains of Alzheimer’s disease (AD) patients. For many years, the mainstream view on the pathogenesis of AD believes that β-amyloid (Aβ) usually acts independently in addition to triggering functions. However, the evidence now accumulating indicates another case that these pathological types have synergies. The objective of this study was to investigate whether effects of Aβ pathology on cognition were mediated by AD pathologies, including tau-related pathology (p-tau), neurodegeneration (t-tau, MRI measurements), axonal injury (NFL), synaptic dysfunction (neurogranin), and neuroinflammation (sTREM2, YKL-40).

**Methods:**

Three hundred seventy normal controls (CN) and 623 MCI patients from the ADNI (Alzheimer’s Disease Neuroimaging Initiative) database were recruited in this research. Linear mixed-effects models were used to evaluate the associations of baseline Aβ with cognitive decline and biomarkers of several pathophysiological pathways. Causal mediation analyses with 10,000 bootstrapped iterations were conducted to explore the mediation effects of AD pathologies on cognition.

**Results:**

Tau-related pathology, neurodegeneration, neuroinflammation are correlated with the concentration of Aβ, even in CN participants. The results show that age, gender, and *APOE ε4* carrier status have a moderating influence on some of these relationships. There is a stronger association of Aβ with biomarkers and cognitive changes in the elderly and females. In CN group, Aβ pathology is directly related to poor cognition and has no mediating effect (*p* < 0.05). In mild cognitive impairment, tau-related pathology (26.15% of total effect) and neurodegeneration (14.8% to 47.0% of total effect) mediate the impact of Aβ on cognition.

**Conclusions:**

In conclusion, early Aβ accumulation has an independent effect on cognitive decline in CN and a tau, neurodegeneration-dependent effect in the subsequent cognitive decline in MCI patients.

**Supplementary Information:**

The online version contains supplementary material available at 10.1186/s13195-021-00943-z.

## Background

An essential pathology of Alzheimer’s disease (AD) is the gradual aggregation of β-amyloid (Aβ) in the brain, a process that begins decades before cognitive symptoms appear. The detection of abnormal Aβ accumulation may support the clinical diagnosis of AD [1–3]. For many years, it has been generally believed that changes of Aβ promote the progression of AD and trigger harmful cascade reactions, including tau pathology and neurodegeneration. Except this trigger function, it is generally believed that Aβ and tau act independently without a specific interaction. However, there is now accumulating evidence showing that this is not the case, and the two pathologies may have a synergistic effect [4]. In addition to Aβ aggregation, downstream pathological processes are also shown to play key roles in AD progression. Reducing Aβ showed cognitive benefits in AD mouse models but failed to improve the clinical symptoms of AD patients in many clinical trials. Perhaps the simplest explanation is that those AD mouse models only have plaque pathology, while other pathologies may also exist in the cerebrum of AD patients, such as axonal injury, synaptic dysfunction, and neuroinflammation [5–7]. Therefore, Aβ is a necessary but not a sufficient condition for AD [8]. AD may be caused not only by the pathology of Aβ and tau but also by the synergy and interaction among various pathological processes, which subsequently leads to cognitive decline.

The objectives of our study were (1) to investigate whether Aβ pathology is related to downstream pathophysiological processes and cognitive levels; (2) to explore whether the major unchangeable AD risk factors such as age, gender, and APOE ε4 status regulate these associations; and (3) to investigate to what extent those associations represent particular downstream changes related to Aβ or to what extent they are driven by other relevant biomarkers. Advances in developing new cerebrospinal fluid (CSF) or blood biomarkers provide insights into tracking pathological processes [6, 9–12]. Research into the synergistic interactions between the biomarkers of AD pathology will facilitate the understanding and the prevention of AD.

To implement these objectives, we investigated biomarkers that reflect the pathophysiology of AD, including tau-related pathology (phosphorylated tau, p-tau), neurodegeneration (total tau, t-tau; MRI measurements), axonal injury (neurofilament light, NFL), synaptic dysfunction (neurogranin), and neuroinflammation (soluble triggering receptor on myeloid cells 2, sTREM2; YKL-40) [13]. All those analyses were conducted among non-demented individuals.

## Methods

### Participants

The data used in the study was acquired from the Alzheimer’s Disease Neuroimaging Initiative (ADNI) database. We included 993 subjects with available basic clinical characteristics, cerebrospinal fluid data, imaging data, and cognitive assessment data from the ADNI database. The ADNI database classified subjects clinically as cognitively normal (CN, MMSE > 24, CDR = 0), mild cognitive impairment (MCI; MMSE > 24, CDR = 0.5) or AD dementia following predefined criteria [14]. Individuals with subjective memory complaints at baseline were not excluded from the analyses. Instead, they were included within the CN group.

### Measurements of CSF and plasma biomarkers

Those CN and MCI subjects with available baseline CSF Aβ_42_, p-tau, t-tau, sTREM2, YKL-40, plasma NFL, and MRI information were included in the analysis. The methods and analyses for biomarkers and imaging have been previously described in detail [15]. CSF Aβ_42_, p-tau, and t-tau levels (unit: pg/mL) were completed at the University of Pennsylvania through the multiple xMAP Luminex platforms (Luminex Corp, Austin, TX, USA) and INNOBIA AlzBio3 kit (Fujirebio, Ghent, Belgium) [16]. CSF sTREM2 measurements (unit: pg/mL) were done with an MSD platform-based assay, previously reported and validated [17]. The CSF YKL-40 levels (unit: ng/mL) were measured by the MicroVue YKL-40 ELISA (Quidel Corp.) at the University of Washington [18]. All CSF biomarker assays were performed in duplicate and averaged. Plasma NFL concentrations (unit: pg/mL) were measured on a Single-molecule array (Simoa) HD-1 analyzer (Quanterix) using an in-house immunoassay [19]. *APOE* genotype was determined by genotyping two single-nucleotides (*rs429358*, rs7412) [20]. Genotype was analyzed as a dichotomous variable. The subject is classified as an *APOE ε4* carrier if carrying at least one *APOE ε4* allele.

### MRI assessment

The protocol of the ADNI FreeSurfer-based pipeline is available online (http://adni.loni.usc.edu/) and in previous publications [21]. The MRI T1-weighted image underwent initial preprocessing, intensity normalization, and gradient expansion. After a hybrid watershed/surface deformation removed the non-brain tissue, the volume structures of subcortical white matter and deep gray matter were segmented by automatic Talairach transform. The volume of the whole brain, hippocampus, entorhinal, and mid temporal was extracted as the regions of interest (ROI).

### Cognitive assessment

We downloaded the episodic memory (MEM) and executive function (EF) comprehensive scores, as well as the recently verified language (LAN) and visual-spatial functions (VS) from the website as tools to track the trajectory of cognitive measurement. These scores are extracted from ADNI neuropsychological tests, which are comprehensive scores after optimization of psychological measurement. These measurements have been verified before, proving robust and externally effective [22, 23].

### Statistical analyses

All statistical analyses were performed in R v.3.6.3. Outliers of baseline CSF Aβ_42_ concentrations are considered as three standard deviations (SD) higher or lower than the overall population means. These subjects (*n* = 6) were excluded. Baseline characteristics were compared between diagnostic groups using Student’s *t*-tests or Wilcoxon rank-sum tests for continuous and *χ*^2^-tests for categorical measures. The first aim of this study was to measure the direct associations of CSF Aβ_42_ with cognitive measurements and various available biomarkers. To this end, we used each biomarker and cognitive assessment results as the dependent variable of interest and the CSF Aβ_42_ level as the independent variable in the multivariate linear regression model. To determine the association of CSF Aβ_42_ levels with biomarkers and cognitive ability changes, we utilized a pre-established method in which we fitted linear mixed-effects (LME) models with various measurements as the dependent variable and time as the independent variable, controlling for random slope and intercept. The LME model was also used to simulate the rate of changes of various measurements for subsequent analyses. We added a new variable resulting from the product of risk factors and CSF Aβ_42_ to the model to evaluate the interaction effects of CSF Aβ_42_ levels and main AD risk elements (i.e., age, gender, and *APOE ε4* status) on each indicator.

We used various biomarkers as mediators for our main hypothesis to analyze the mediation between CSF Aβ_42_ and multiple cognitive measures. The purpose of this analysis is to assess whether the previously discovered association between CSF Aβ_42_ and cognition is partially, completely, or not mediated by AD pathology. In each model, CSF Aβ_42_ values were included as an independent variable, cognitive measurements as the dependent variable. All mediational tests were performed with 10,000 bootstrap replications. The cut-off value of biomarker which used to indicate normal (negative) and abnormal (positive) might be considered as a characteristic for the existence of Aβ pathology [24]. The cut-off concentration of CSF Aβ_42_ is 192 pg/ml. This cut-off value was used to divide the participants into A− and A+ groups for subsequent subgroup analyses.

All analyses were adjusted age, sex, *APOE ε4* carrying status, and educational level as covariates, and additionally adjusted intracranial volume during MRI measurements analyses. Since all outcome variables in the model were converted to standardized z-scores, the coefficient refers to the standardized effect.

## Results

The demographics, CSF Aβ, mediators, and neurocognitive data at baseline and follow-up are shown in Table 1 and Additional file 3. A total of 993 individuals (370 CN and 623 MCI) free of dementia were included. As expected, there are differences in cognitive assessment between clinical diagnosis groups (MEM, *p* < 0.001; EF, *p* < 0.001; LAN, *p* < 0.001; VS, *p* < 0.001). The cognitive decline rates of MCI participants are significantly higher than that of CN population (MEM, *p* < 0.001; EF, *p* < 0.001; LAN, *p* < 0.001; VS, *p* < 0.001). There is lower CSF Aβ_42_ (*p* < 0.001) and higher p-tau (*p* = 0.002), t-tau (*p* < 0.001), Neurogranin (*p* < 0.001), and plasma NFL (*p* < 0.001) in the MCI group compared with CN group. We did not find any difference between the two groups in terms of educational level, CSF sTREM2, and CSF YKL-40 concentration, which was closely resembled previous findings of this cohort [25, 26].

**Table 1:** Clinical characteristics of participants in individual groups in the current study

	CN (***n*** = 370)	MCI (***n*** = 623)	***p*** value
Age (years)	73.78 ± 5.91	72.42 ± 7.53	0.012
Gender (F/M)	194/176	256/366	0.001
Education (years)	16.39 ± 2.62	16.05 ± 2.76	0.081
*APOE ε4* carriers (%)	27.84%	49.12%	< 0.001
CSF measures			
Aβ_42_	200.34 ± 50.95	171.70 ± 52.28	< 0.001
p-tau	32.13 ± 18.40	39.07 ± 22.38	< 0.001
t-tau	67.51 ± 32.16	90.56 ± 54.82	< 0.001
Neurogranin	379.95 ± 284.78	508.58 ± 343.28	< 0.001
sTREM2	4104.09 ± 2122.88	4448.60 ± 2285.17	0.508
YKL-40	392.56 ± 127.99	393.71 ± 131.49	0.966
Plasma NFL	34.32 ± 21.96	39.20 ± 22.85	< 0.001
MRI measures			
Whole brain	1040346.1 ± 104238.7	1047243.7 ± 109038.5	0.411
Hippocampus	7467.2 ± 851.0	6867.8 ± 1142.1	< 0.001
Entorhinal	3854.9 ± 610.2	3553.4 ± 724.3	< 0.001
Mid temporal	20342.2 ± 2649.5	19895.1 ± 2860.6	0.051
Cognitive composite measures			
Memory function	1.05 ± 0.57	0.20 ± 0.69	< 0.001
Executive function	0.79 ± 0.82	0.23 ± 0.88	< 0.001
Language	0.81 ± 0.71	0.21 ± 0.77	< 0.001
Visuospatial functioning	0.20 ± 0.60	− 0.06 ± 0.75	< 0.001

Categorical variables are reported as numbers and percentages; continuous variables are reported as means ± SDs

*Abbreviations*: *CN* normal controls, *MCI* mild cognitive impairment, *M* male, *F* female, *APOE ε4* apolipoprotein E4, *CSF* cerebrospinal fluid, *Aβ* amyloid-β, *p-tau* phosphorylated tau, *t-tau* total tau, *sTREM2* soluble triggering receptor on myeloid cells 2, *NFL* neurofilament light, *MRI* magnetic resonance imaging

### Associations of CSF Aβ_42_ with various biomarkers and cognitive measures

**Fig. 1:**
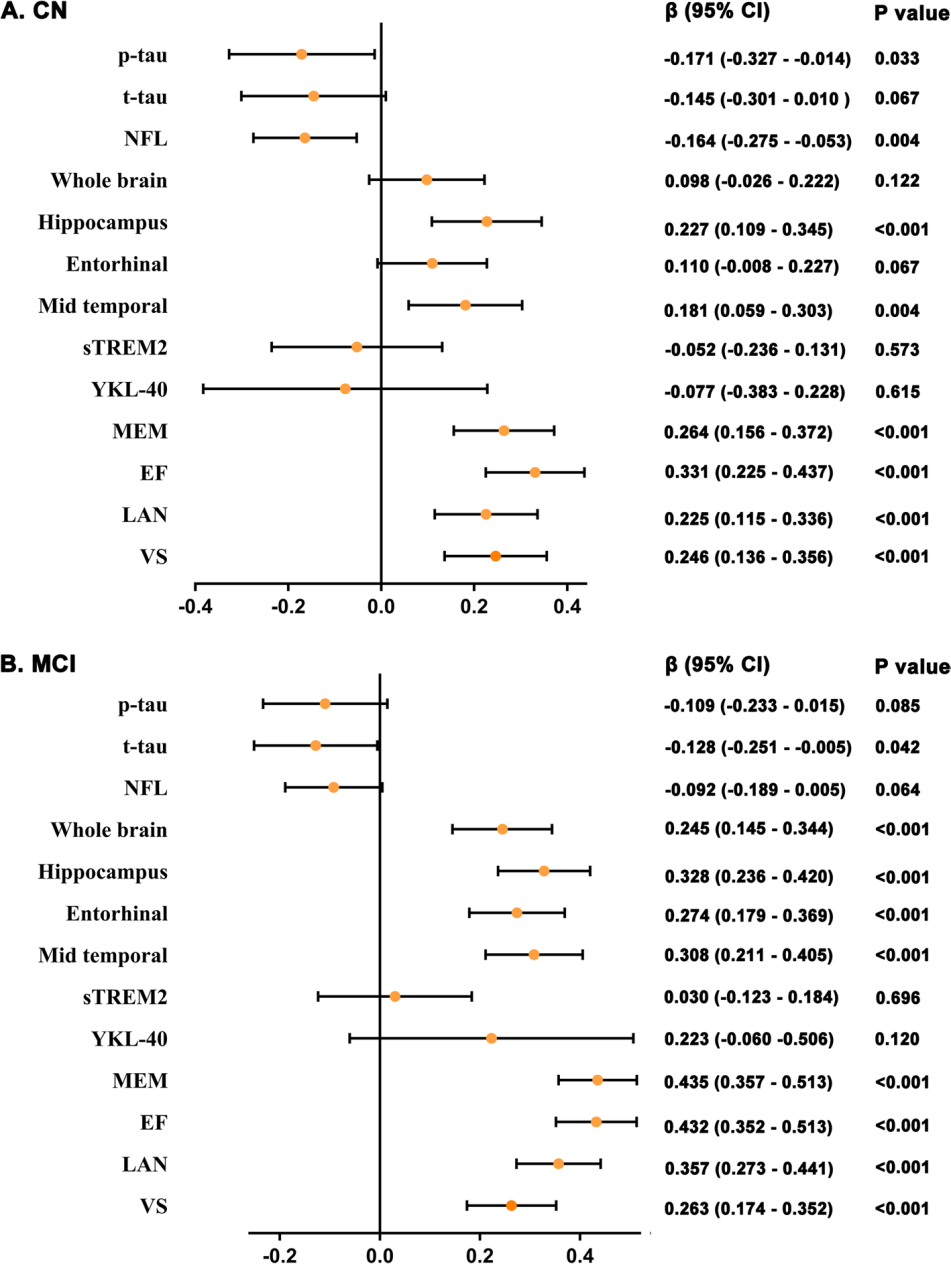
Main effects of Aβ on biomarkers and cognitive measures in non-dementia participants. The figure shows the associations of baseline CSF Aβ42 on longitudinal biomarkers and cognitive measurements in CN and MCI populations, respectively. *p* values were extracted from linear mixed-effects models adjusted for age, sex, *APOE ε4* carrying status, and educational levels. CN, normal controls; MCI mild cognitive impairment; Aβ, amyloid-β; p-tau, phosphorylated tau; t-tau, total tau; NFL, neurofilament light; sTREM2, soluble triggering receptor on myeloid cells 2; MEM, memory function; EF, executive function; LAN, language; VS, visuospatial functioning

In our first main analysis, we calculated the associations of CSF Aβ_42_ with all mediators and cognitive measures. Individuals with lower CSF Aβ_42_ levels had more significant tau pathology, neurodegeneration, and severe synaptic dysfunction, as indicated by CN participants’ CSF p-tau, t-tau, and neurogranin levels (Additional file 4). Similar associations were found in the subsequent longitudinal analyses (Fig. 1). Lower baseline CSF Aβ_42_ level also indicated faster cognitive decline during follow-up (Fig. 1). In the MCI group, CSF Aβ_42_ was closely related to tau pathology, neurodegeneration, synaptic dysfunction, neuroinflammation, and cognitive level (Additional file 5). Similar associations were found in the subsequent longitudinal analyses except for p-tau and neuroinflammation (Fig. 1).

### Age, sex, and APOE ε4 interactions with CSF Aβ_42_ on biomarkers

We only found CSF Aβ_42_ level interacted with sex on p-tau (*p* = 0.011) and VS (longitudinal, *p* = 0.030) in CN participants. No significant interactions with age and *APOE ε4* status were detected using CSF Aβ_42_ as a marker of amyloid pathology (Additional file 4). In MCI group, the first interaction effect tested in the association between CSF Aβ_42_ and other measures was age. We observed that the interaction effect was important for longitudinal p-tau (*p* = 0.016), NFL (*p* = 0.017), MRI (hippocampus, *p* = 0.011; entorhinal, *p* = 0.007; mid temporal, *p* = 0.036), and cognitive measurements (MEM, *p* = 0.008; EF, *p* = 0.003; VS, *p* = 0.014). CSF Aβ_42_ interacted with sex on longitudinal entorhinal volume (*p* = 0.042), MEM (*p* = 0.003), and EF (*p* = 0.015). Regarding the interaction between *APOE ε4* status and CSF Aβ_42_, it was only significant for hippocampus volume (*p* < 0.001) and mid temporal volume (*p* = 0.011) (Additional file 5).

### Causal mediation analyses

**Fig. 2:**
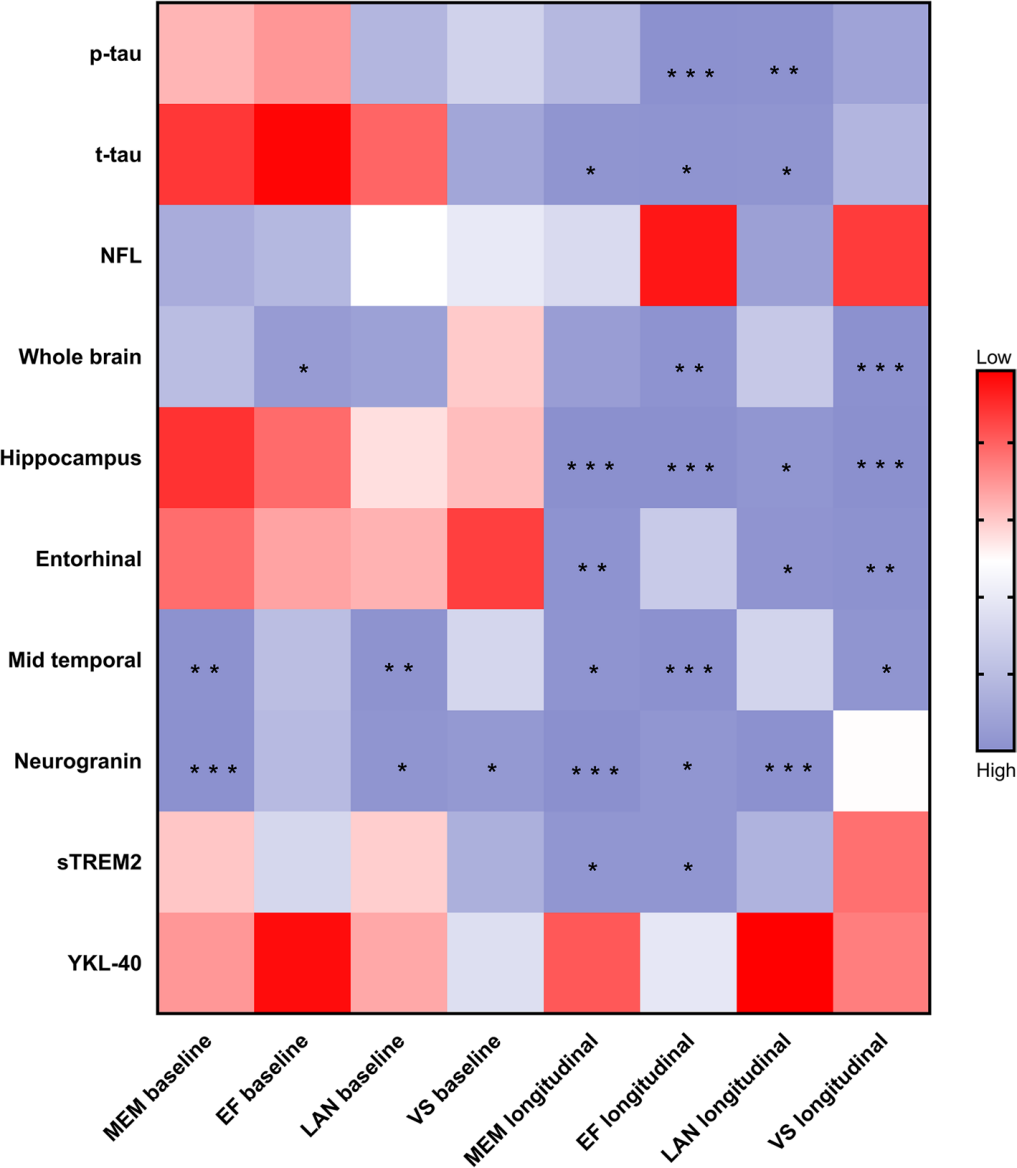
Effects of biomarkers on cognitive composite measures in CN participants. The figure shows the relationship of each biomarker at baseline with baseline and longitudinal cognitive measurements. Meaningful results have been marked with asterisks. *, **, and ***: *p* value< 0.05 and *p* value < 0.01, and *p* value< 0.001, respectively. CN, normal controls; p-tau, phosphorylated tau; t-tau, total tau; NFL, neurofilament light; sTREM2, soluble triggering receptor on myeloid cells 2; MEM, memory function; EF, executive function; LAN, language; VS, visuospatial functioning.

Preliminary regression analyses of different cognitive groups showed the associations of the pathological index with cognitive measurements in the model controlling for age, gender, educational level, and *APOE ε4* status. We tested whether the association between CSF Aβ_42_ and cognitive measurements was mediated by tau pathology and/or neurodegeneration, synaptic dysfunction, neuroinflammation. There is no evidence that CSF Aβ_42_ contributes to cognitive impairments via modulating other AD pathology in CN participants (Additional file 1, 2). However, analyses suggested that tau, neurodegeneration, synaptic dysfunction, and neuroinflammation directly impact cognitive decline (Fig. 2, Additional file 6).

**Fig. 3:**
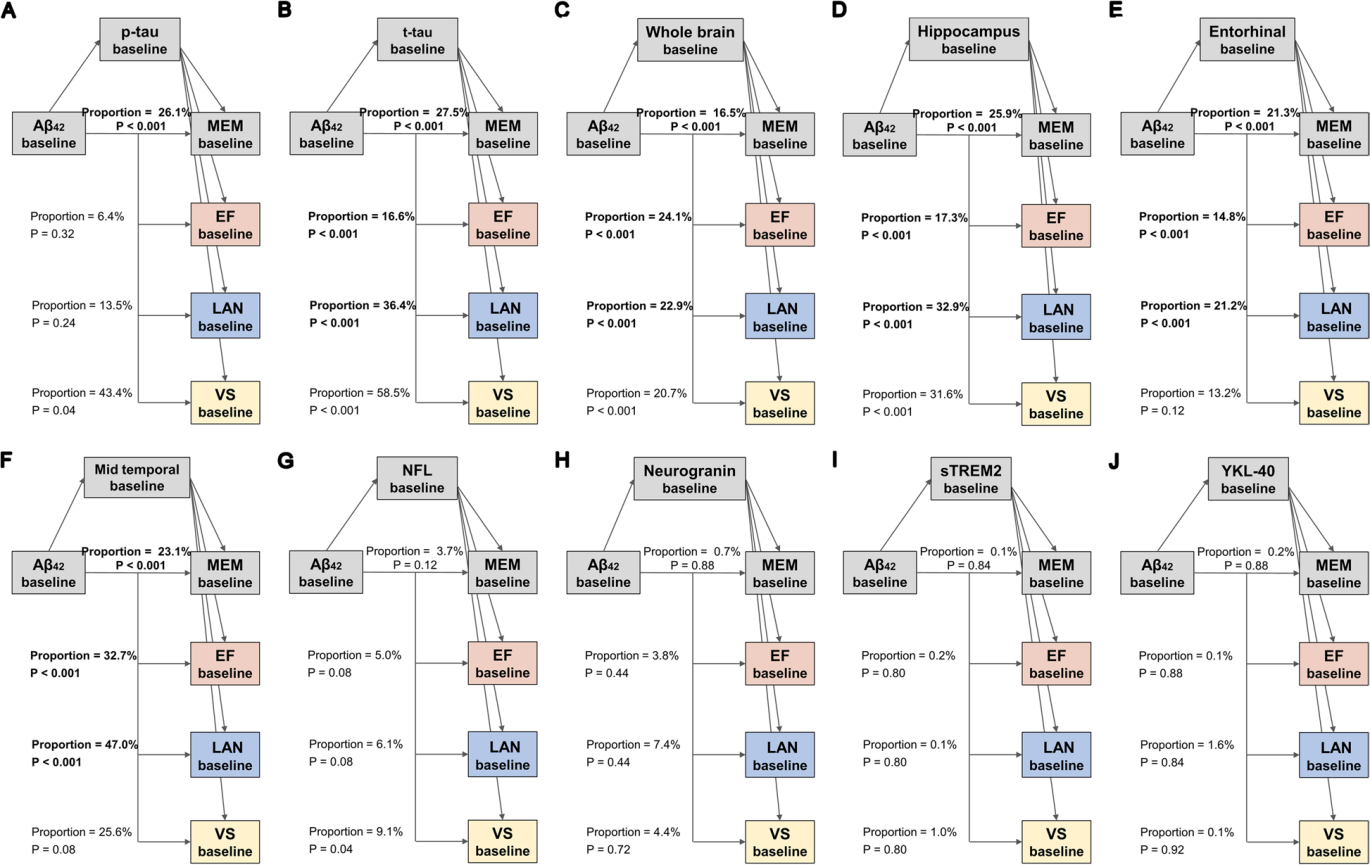
Mediation analyses of Aβ and baseline cognition with biomarkers as mediators in MCI. The bold *p* values indicate the mediation pathways are meaningful among the MCI participants. The proportions shown in the figure indicate the proportion of mediating factors in the total effect of amyloid pathology on cognition. MCI, mild cognitive impairment; Aβ, amyloid-β; p-tau, phosphorylated tau; t-tau, total tau; NFL, neurofilament light; sTREM2, soluble triggering receptor on myeloid cells 2; MEM, memory function; EF, executive function; LAN, language; VS, visuospatial functioning

**Fig. 4:**
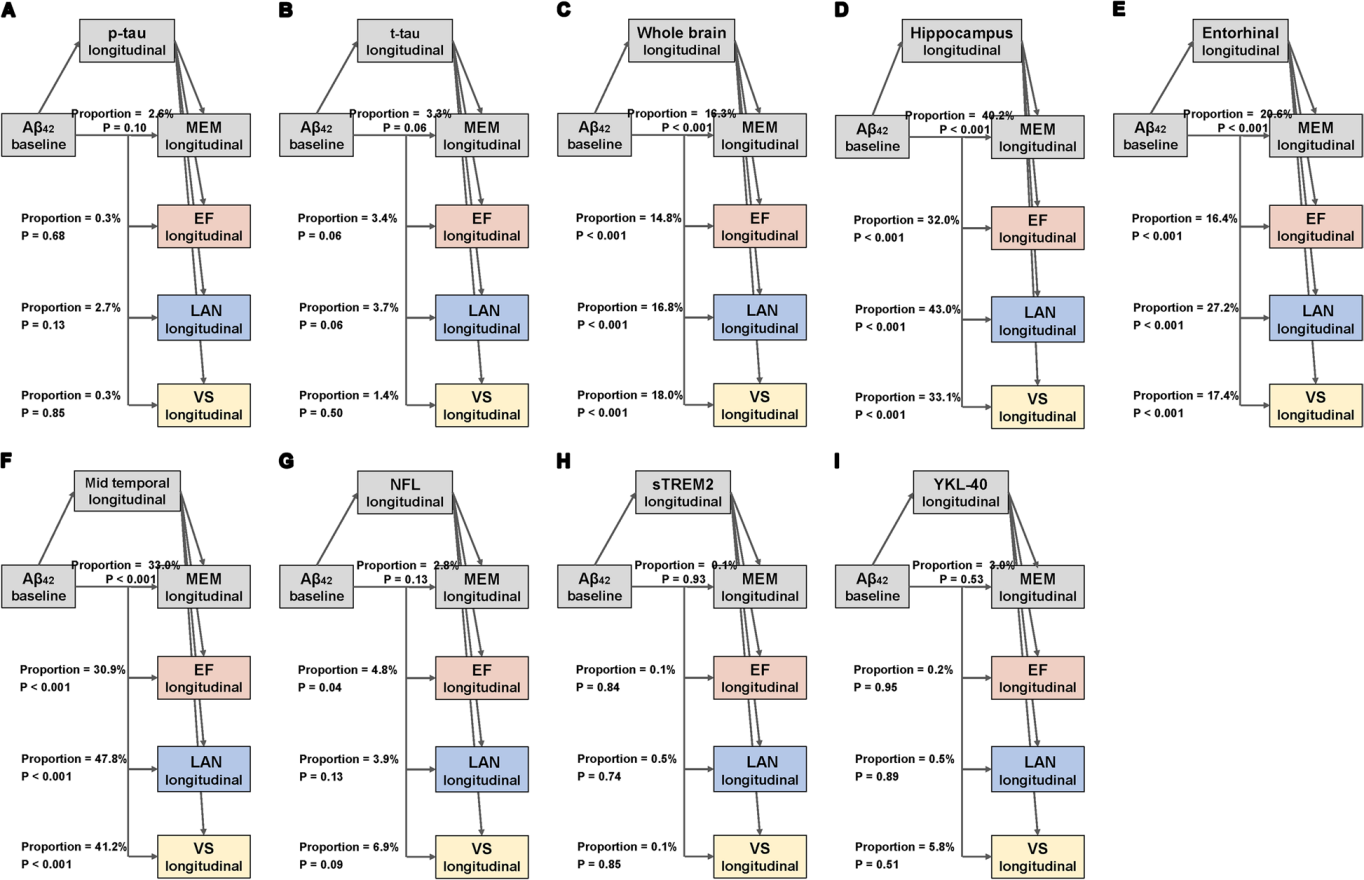
Mediation analyses of Aβ and longitudinal cognition with biomarkers as mediators in MCI. The bold *p* values indicate the mediation pathways are meaningful among the MCI participants. The proportions shown in the figure indicate the proportion of mediating factors in the total effect of amyloid pathology on cognition. MCI, mild cognitive impairment; Aβ, amyloid-β; p-tau, phosphorylated tau; t-tau, total tau; NFL, neurofilament light; sTREM2, soluble triggering receptor on myeloid cells 2; MEM, memory function; EF, executive function; LAN, language; VS, visuospatial functioning

The direct impact of tau pathology, neurodegeneration, and synaptic dysfunction on cognition was also observed in the MCI participants. Figures 3 and 4 show the final models of MCI participants in which some mediators showed at least a trend to significance in the mediation path. First, we found that the relationship between Aβ pathology and cognitive impairment was partially mediated by baseline tau pathology (CSF p-tau, 26.15% of total effect) and neurodegeneration (CSF t-tau, 16.6% to 36.4% of total effect; whole brain volume, 16.5% to 24.1% of total effect; hippocampus volume, 17.3% to 32.9% of total effect; entorhinal volume, 14.8% to 21.3% of total effect; and mid temporal volume, 23.1% to 47.0% of total effect) (Fig. 3). Figure 4 illustrates the results of mediation using the longitudinal change of the biomarkers as mediating variables. Similarly, the effect of amyloid on cognitive decline was partly mediated by longitudinal neurodegeneration (whole brain volume, 16.8% to 18.0% of total effect; hippocampus volume, 32.0% to 49.2% of total effect; entorhinal volume, 16.4% to 29.6% of total effect; and mid temporal volume, 30.9% to 47.8% of total effect).

Although the results of this study show a particular association between neuroinflammation and cognitive decline, we have not observed a mediating effect (Additional file 7). Given that a significant positive correlation between Aβ and cognitive measures exists, we conducted secondary analyses that categorized the sample into four subgroups: A-CN group, A+CN group, A-MCI group, and A+MCI group (Additional file 8, 9, 10 and 11). The above results were replicated in the A + MCI group (Additional file 11).

## Discussion

In this study, we investigated the associations of Aβ pathology with cognitive changes and biomarkers which represent the downstream pathophysiological processes of Aβ in a cohort of non-demented participants. Our results showed that the concentration of Aβ was negatively correlated with tau-related pathology, neurodegeneration, and neuroinflammation, even in CN participants, suggesting that a reduction in Aβ could slow its downstream pathophysiological processes. Previous studies showed that anti-oligomeric Aβ antibodies effectively reduced plaque load, tau hyperphosphorylation, and microglial activation, and these antibodies improved the cognitive ability in mouse models. These may be the protective effects of anti-oligomeric Aβ antibodies before cognitive decline [27]. We also found age, gender, and *APOE ε4* carrier status had moderating effects on some of these correlations. The interaction analyses showed there were stronger associations of Aβ with cognitive changes and biomarkers in the elderly and females. In the mediation analysis, most *p* values with statistical significance were less than 0.001. This means that even the FDR correction is not likely to affect the final conclusion.

We conducted a series of mediation analyses to explore which factors mediate the impact of Aβ on cognition. In the CN group, Aβ, tau pathology, neurodegeneration, axonal injury, synaptic dysfunction, and neuroinflammation were directly related to cognitive decline without mediation effects. Several previous studies reported that people with decreased Aβ levels had a faster cognitive decline in memory and executive function [28–31]. But so far, there are relatively limited studies evaluating the relationship between Aβ and the longitudinal trajectories of cognitive performance. Our results are consistent with a recent finding from longitudinal cohort studies that Aβ and tau were associated with each other even in the CN population [32–35]. Aβ, tau, neurodegeneration, synaptic dysfunction, and neuroinflammation all seem to directly associate with cognitive decline and contribute to memory decline in the preclinical stage of AD [36, 37]. This may explain why anti-Aβ therapy alone brings limited benefits in slowing down the rate of progression. Of course, in addition to causing downstream pathological processes, Aβ may also lead to cognitive impairment in an independent manner.

In MCI participants, the effects of Aβ on cognitive measurements were partially mediated by tau pathology and neurodegeneration. Accumulating evidence has shown that upstream Aβ accumulation is related to abnormal changes in downstream pathological biomarkers, including abnormal tau, neurological dysfunction, glial activation, neuron loss, and brain atrophy, which is consistent with our result [38]. However, whether Aβ accumulation is sufficient to trigger the pathological cascades of AD and ultimately lead to cognitive impairment and dementia remains to be confirmed. Several studies have investigated a prediction model for a cascade of sequential reactions [39–41]. We found that abnormal Aβ and tau exhibited synergistic effects, leading to memory decline in the MCI population. The mediation analysis indicated that Aβ might also affect cognition via tau pathology and neurodegeneration. Consistent with our finding, the existing evidence strongly supports the key role of pathological Aβ accumulation in mediating the pathogenesis of AD. The mechanism may not be simple as we initially expected. However, the main difficulty in deterministic verification is that there is no dataset with a long enough follow-up to monitor longitudinal changes because it is assumed that the process of brain Aβ takes decades [42]. The role of Aβ in AD needs to be further clarified in the further research.

Previous mediation studies did not include all the pathological factors [39, 40]. Two neuroinflammation markers, one of which is related to microglial activation (sTREM2), correlate with cognition. However, we have not observed its role as a mediator. sTREM2 is the soluble extracellular domain of the TREM2 receptor, which is mainly expressed in the microglia of the central nervous system. Tau inhibition reduces the upregulation of inflammatory markers expressed by microglia, which suggests that tau may help increase the inflammatory response nominally attributed to Aβ. It is inferred that microglia may serve as a potential mediator factor of Aβ-tau synergic interaction, which needs further research [25]. The evidence shows that Aβ enhances the effect of microglial activation on tau protein diffusion. When these three pathologies coexist in the human brain, they will synergistically interact and jointly determine the development of dementia [43]. Future studies are still warranted to elucidate the relationships of amyloid pathology and neuroinflammation with cognitive decline.

In addition, we only observed the direct effects of tau pathology on cognitive changes in A + individuals. Contrary to the opinion that there is no particular interaction between Aβ and tau except for the former’s triggering function, there is now evidence that functions of Aβ may be more complex [44]. Some evidence suggests that pathological progression of tau protein in AD may require the deposition of Aβ. Human neuropathology studies have shown that tau pathology usually does not progress from the entorhinal cortex to the neocortex, and amyloid pathology does not occur simultaneously [33, 45–47]. A study of CSF biomarkers in individuals aged 50 to 90 years old also demonstrated a synergistic interaction between Aβ and tau in predicting longitudinal memory decline. In this relationship, t-tau and p-tau levels were associated with cognitive ability only in the presence of Aβ deposition [48]. This further confirms that the presence of Aβ enhances tau pathology. It can be inferred that the most effective method for delaying AD may be the combination of anti-Aβ and anti-tau therapies. In addition, tau protein is more stable in the presence of Aβ, which makes it has a longer half-life and higher biological activity. Therefore, based on the inverse relationship between the tau conversion rate and the existence of Aβ, we can implement co-treatment in the early stage of the disease, since anti-Aβ treatment will in turn therapeutically enhance the clearance of tau [49].

## Limitations

There were still some possible limitations in our research. Firstly, since this study is a single-center study, the results still need to be verified in larger longitudinal cohorts. Secondly, ADNI has a relatively pure AD population because it mainly includes MCI patients. Reproduction of findings from different AD phenotypes and participants from other cohorts will be helpful. Thirdly, the relationships between Aβ and cognitive decline still need to be further explored. In several previous studies, Aβ_42_ was expressed in a ratio to Aβ_40_ to assess the pathologic species while accounting for individual differences in amyloid production [50, 51]. We also considered this issue in the design stage of this study, because this ratio was reported as a possible better predictor of brain Aβ deposition. However, considering the possible deviation caused by inconsistent measurement methods and the sample sizes, we finally used Aβ_42_ as a marker for Aβ pathology. Future researches may consider using ratios to explore whether there are differences in research results.

## Conclusions

In conclusion, our research found associations of Aβ pathology with cognition and several AD pathologies, including tau-related pathology, neurodegeneration, axonal injury, synaptic dysfunction, and neuroinflammation. Though the underlying mechanisms were not completely clear, these results still offer new evidence for the synergistic effect among pathological processes. Early Aβ accumulation has an independent impact on cognitive decline and a tau, neurodegeneration-dependent effect in the subsequent cognitive decline. Our results need to be repeated in large samples, and the underlying mechanism needs to be explored in further research.

## Supplementary Information

**Additional file 1.** Mediation analyses of Aβ and baseline cognitive measurements with biomarkers as mediators in CN participants.

**Additional file 2.** Mediation analyses of Aβ and rates of cognitive measurements with biomarkers as mediators in CN participants.

**Additional file 3.** Longitudinal clinical characteristics of participants in individual groups in the current study.

**Additional file 4.** Main and interactions effects of Aβ on biomarkers and cognitive measures in CN participants.

**Additional file 5.** Main and interactions effects of Aβ on biomarkers and cognitive measures in MCI participants.

**Additional file 6.** Effects of biomarkers on cognitive composite measures in CN participants.

**Additional file 7.** Effects of biomarkers on cognitive composite measures in MCI participants.

**Additional file 8.** Mediation analyses of Aβ and cognitive measurements with biomarkers as mediators in A-CN participants.

**Additional file 9.** Mediation analyses of Aβ and cognitive measurements with biomarkers as mediators in A+CN participants.

**Additional file 10.** Mediation analyses of Aβ and cognitive measurements with biomarkers as mediators in A-MCI participants.

**Additional file 11.** Mediation analyses of Aβ and cognitive measurements with biomarkers as mediators in A+MCI participants.

## Data Availability

The dataset generated and analyzed in the current study is available from the corresponding author on reasonable request.

## Abbreviations

AD: Alzheimer’s disease
CSF: Cerebrospinal fluid
CN: Normal controls
MCI: Mild cognitive impairment
M: Male
F: Female
*APOE ε4*: Apolipoprotein E4
CSF: Cerebrospinal fluid
Aβ: Amyloid-β
p-tau: Phosphorylated tau
t-tau: Total tau
sTREM2: Soluble triggering receptor on myeloid cells 2
NFL: Neurofilament light
MRI: Magnetic resonance imaging
MEM: Memory function
EF: Executive function
LAN: Language
VS: Visuospatial functioning
