# Supplementary material for: P-tau and neurodegeneration mediate the effect of β-amyloid on cognition in non-demented elders
Source: Alzheimers Res Ther. 2021 Dec 15;13:200. doi: 10.1186/s13195-021-00943-z (PMC8675473; doi:10.1186/s13195-021-00943-z)
Supplement: Supplementary file 2 — Additional file 2. Mediation analyses of Aβ and rates of cognitive measurements with biomarkers as mediators in CN participants. [file 13195_2021_943_MOESM2_ESM.docx]

**
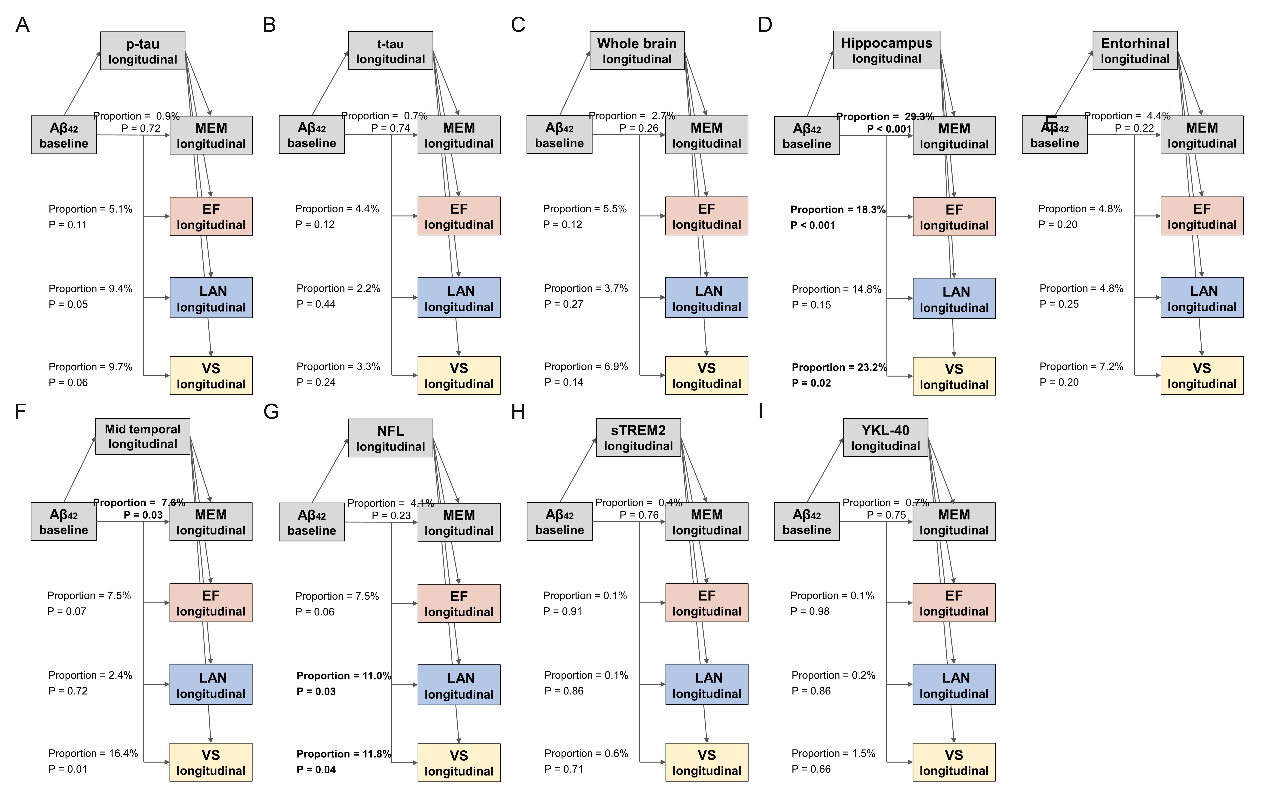
** **Additional file 2.** Mediation analyses of Aβ and rates of cognitive measurements with biomarkers as mediators in CN participants.

Abbreviations: CN, Normal controls; Aβ, Amyloid-β; p-tau, Phosphorylated tau; t-tau, Total tau; NFL, Neurofilament light; sTREM2, Soluble triggering receptor on myeloid cells 2; MEM, Memory function; EF, Executive function; LAN, Language; VS, Visuospatial functioning.
