# Supplementary material for: P-tau and neurodegeneration mediate the effect of β-amyloid on cognition in non-demented elders
Source: Alzheimers Res Ther. 2021 Dec 15;13:200. doi: 10.1186/s13195-021-00943-z (PMC8675473; doi:10.1186/s13195-021-00943-z)
Supplement: Supplementary file 3 — Additional file 3. Longitudinal clinical characteristics of participants in individual groups in the current study. [file 13195_2021_943_MOESM3_ESM.docx]

**Additional file 3.** longitudinal clinical characteristics of participants in individual groups in the current study.

|  | CN | |  | MCI | |  | P value |
| --- | --- | --- | --- | --- | --- | --- | --- |
|  | n | means ± SD |  | n | means ± SD |  |  |
| CSF measures |  |  |  |  |  |  |  |
| Aβ_42_ | 182 | -2.154±3.031 |  | 349 | -1.917±1.994 |  | 0.873 |
| p-tau | 182 | 2.662±1.720 |  | 349 | 3.285±2.187 |  | 0.001 |
| t-tau | 182 | 1.414±1.967 |  | 349 | 2.023±2.673 |  | 0.023 |
| Neurogranin | - | - |  | - | - |  | - |
| sTREM2 | 129 | 181.223±68.609 |  | 237 | 185.749±70.005 |  | 0.566 |
| YKL-40 | 54 | 4.282±8.719 |  | 75 | 6.000±7.931 |  | 0.086 |
| Plasma NFL | 291 | 2.315±1.606 |  | 448 | 2.821±2.346 |  | 0.011 |
| MRI measures |  |  |  |  |  |  |  |
| Whole brain | 295 | -7971.46±3617.81 |  | 474 | -9671.9±5099.509 |  | <0.001 |
| Hippocampus | 295 | -108.134±52.853 |  | 474 | -145.176±76.853 |  | <0.001 |
| Entorhinal | 295 | -53.634±30.258 |  | 474 | -71.130±38.226 |  | <0.001 |
| Mid temporal | 295 | -241.291±159.213 |  | 474 | -339.136±249.781 |  | <0.001 |
| Cognitive measures |  |  |  |  |  |  |  |
| Memory function | 362 | -0.041±0.069 |  | 600 | -0.113±0.108 |  | <0.001 |
| Executive function | 362 | -0.055±0.076 |  | 600 | -0.121±0.123 |  | <0.001 |
| Language | 362 | -0.051±0.079 |  | 600 | -0.139±0.152 |  | <0.001 |
| Visuospatial functioning | 362 | -0.044±0.052 |  | 600 | -0.072±0.0740 |  | <0.001 |

Categorical variables are reported as numbers and percentages; continuous variables are reported as means ± SDs.

**Abbreviations:** CN, Normal controls; MCI, Mild cognitive impairment; CSF, Cerebrospinal fluid; Aβ, Amyloid-β; p-tau, Phosphorylated tau; t-tau, Total tau; sTREM2, Soluble triggering receptor on myeloid cells 2; NFL, Neurofilament light; MRI, Magnetic resonance imaging.
