# Supplementary material for: P-tau and neurodegeneration mediate the effect of β-amyloid on cognition in non-demented elders
Source: Alzheimers Res Ther. 2021 Dec 15;13:200. doi: 10.1186/s13195-021-00943-z (PMC8675473; doi:10.1186/s13195-021-00943-z)
Supplement: Supplementary file 4 — Additional file 4. Main and interactions effects of Aβ on biomarkers and cognitive measures in CN participants. [file 13195_2021_943_MOESM4_ESM.docx]

**Additional file 4.** Main and interactions effects of Aβ on biomarkers and cognitive measures in CN participants.

|  | **Main effect** | | **Age interaction** | | **Sex interaction** | | ***APOE ε4* interaction** | |
| --- | --- | --- | --- | --- | --- | --- | --- | --- |
|  | **β** | **P** | **β** | **P** | **β** | **P** | **β** | **P** |
| **Baseline** | | | | | | | | |
| p-tau | **-0.299** | **<0.001** | -0.064 | 0.322 | **0.243** | **0.011** | -0.124 | 0.275 |
| t-tau | **-0.148** | **0.008** | -0.090 | 0.175 | 0.033 | 0.739 | 0.038 | 0.748 |
| NFL | -0.021 | 0.706 | 0.115 | 0.090 | -0.160 | 0.113 | -0.031 | 0.798 |
| Whole brain | -0.004 | 0.899 | 0.022 | 0.584 | 0.118 | 0.051 | 0.039 | 0.587 |
| Hippocampus | 0.052 | 0.316 | -0.017 | 0.792 | 0.112 | 0.242 | 0.061 | 0.594 |
| Entorhinal | 0.010 | 0.849 | 0.064 | 0.345 | 0.084 | 0.406 | 0.068 | 0.572 |
| Mid temporal | 0.003 | 0.956 | 0.053 | 0.380 | 0.029 | 0.743 | 0.094 | 0.374 |
| Neurogranin | **-0.245** | **<0.001** | 0.111 | 0.134 | 0.119 | 0.285 | -0.240 | 0.085 |
| sTREM2 | **0.158** | **0.011** | -0.080 | 0.294 | -0.062 | 0.577 | -0.035 | 0.786 |
| YKL-40 | -0.025 | 0.868 | -0.439 | 0.082 | -0.217 | 0.451 | -0.453 | 0.205 |
| MEM | 0.062 | 0.229 | 0.013 | 0.828 | 0.003 | 0.977 | 0.152 | 0.160 |
| EF | **0.129** | **0.014** | -0.032 | 0.612 | 0.028 | 0.763 | 0.120 | 0.276 |
| LAN | 0.012 | 0.831 | -0.008 | 0.906 | -0.012 | 0.905 | 0.220 | 0.059 |
| VS | -0.061 | 0.291 | 0.056 | 0.410 | -0.169 | 0.099 | -0.066 | 0.589 |
| **Longitudinal** | | | | | | | | |
| p-tau | **-0.171** | **0.033** | -0.192 | 0.069 | -0.080 | 0.589 | 0.223 | 0.197 |
| t-tau | -0.145 | 0.067 | 0.097 | 0.357 | -0.160 | 0.278 | 0.115 | 0.506 |
| NFL | **-0.164** | **0.004** | -0.009 | 0.899 | -0.190 | 0.065 | 0.074 | 0.534 |
| Whole brain | 0.098 | 0.122 | -0.096 | 0.225 | -0.130 | 0.267 | -0.057 | 0.682 |
| Hippocampus | **0.227** | **<0.001** | -0.052 | 0.486 | -0.019 | 0.863 | 0.079 | 0.549 |
| Entorhinal | 0.110 | 0.067 | 0.058 | 0.436 | 0.056 | 0.614 | 0.063 | 0.635 |
| Mid temporal | **0.181** | **0.004** | 0.011 | 0.891 | -0.065 | 0.574 | -0.027 | 0.841 |
| Neurogranin | - | - | - | - | - | - | - | - |
| sTREM2 | -0.052 | 0.573 | 0.096 | 0.437 | -0.071 | 0.687 | 0.042 | 0.832 |
| YKL-40 | -0.077 | 0.615 | 0.262 | 0.299 | -0.165 | 0.566 | 0.207 | 0.565 |
| MEM | **0.264** | **<0.001** | 0.051 | 0.439 | -0.149 | 0.137 | 0.110 | 0.351 |
| EF | **0.331** | **<0.001** | -0.039 | 0.549 | 0.014 | 0.883 | 0.120 | 0.297 |
| LAN | **0.225** | **<0.001** | 0.121 | 0.073 | 0.133 | 0.190 | -0.032 | 0.792 |
| VS | **0.246** | **<0.001** | 0.126 | 0.061 | **0.220** | **0.030** | 0.040 | 0.739 |

Significant effects (P <0.05) are shown in bold. Models included age, sex, education, *APOEε4* status and intracranial volume as covariates.

**Abbreviations:** CN, Normal controls; *APOEε4*, Apolipoprotein E4; Aβ, Amyloid-β; p-tau, Phosphorylated tau; t-tau, Total tau; NFL, Neurofilament light; sTREM2, Soluble triggering receptor on myeloid cells 2; MEM, Memory function; EF, Executive function; LAN, Language; VS, Visuospatial functioning.
