# Supplementary material for: P-tau and neurodegeneration mediate the effect of β-amyloid on cognition in non-demented elders
Source: Alzheimers Res Ther. 2021 Dec 15;13:200. doi: 10.1186/s13195-021-00943-z (PMC8675473; doi:10.1186/s13195-021-00943-z)
Supplement: Supplementary file 5 — Additional file 5. Main and interactions effects of Aβ on biomarkers and cognitive measures in MCI participants. [file 13195_2021_943_MOESM5_ESM.docx]

**Additional file 5.** Main and interactions effects of Aβ on biomarkers and cognitive measures in MCI participants.

|  | **Main effect** | | **Age interaction** | | **Sex interaction** | | ***APOE ε4* interaction** | |
| --- | --- | --- | --- | --- | --- | --- | --- | --- |
|  | **β** | **P** | **β** | **P** | **β** | **P** | **β** | **P** |
| **Baseline** | | | | | | | | |
| p-tau | **-0.468** | **<0.001** | 0.035 | 0.407 | **0.197** | **0.005** | -0.154 | 0.055 |
| t-tau | **-0.373** | **<0.001** | 0.045 | 0.291 | **0.254** | **<0.001** | **-0.244** | **0.002** |
| NFL | -0.076 | 0.088 | -0.014 | 0.764 | 0.067 | 0.378 | 0.041 | 0.628 |
| Whole brain | **0.101** | **0.001** | **-0.112** | **0.001** | -0.052 | 0.322 | -0.021 | 0.722 |
| Hippocampus | **0.192** | **<0.001** | **-0.131** | **0.007** | -0.050 | 0.517 | 0.128 | 0.140 |
| Entorhinal | **0.184** | **<0.001** | -0.003 | 0.958 | -0.011 | 0.893 | 0.023 | 0.809 |
| Mid temporal | **0.204** | **<0.001** | **-0.107** | **0.022** | -0.130 | 0.087 | 0.074 | 0.390 |
| Neurogranin | **-0.227** | **0.003** | 0.140 | 0.094 | 0.083 | 0.579 | -0.142 | 0.365 |
| sTREM2 | **0.126** | **0.012** | **-0.127** | **0.018** | **0.214** | **0.018** | **-0.245** | **0.004** |
| YKL-40 | 0.017 | 0.900 | 0.138 | 0.366 | 0.140 | 0.614 | -0.651 | 0.155 |
| MEM | **0.268** | **<0.001** | **-0.170** | **<0.001** | **-0.167** | **0.020** | 0.152 | 0.061 |
| EF | **0.230** | **<0.001** | 0.022 | 0.623 | -0.119 | 0.105 | -0.160 | 0.055 |
| LAN | **0.151** | **0.001** | -0.002 | 0.969 | -0.122 | 0.103 | -0.028 | 0.745 |
| VS | 0.090 | 0.054 | 0.043 | 0.373 | -0.091 | 0.252 | -0.134 | 0.137 |
| **Longitudinal** | | | | | | | | |
| p-tau | -0.109 | 0.085 | **-0.155** | **0.016** | -0.140 | 0.201 | -0.001 | 0.996 |
| t-tau | **-0.128** | **0.042** | -0.084 | 0.189 | 0.040 | 0.712 | 0.042 | 0.742 |
| NFL | -0.092 | 0.064 | **0.125** | **0.017** | 0.074 | 0.386 | -0.010 | 0.923 |
| Whole brain | **0.245** | **<0.001** | -0.003 | 0.953 | 0.029 | 0.742 | 0.165 | 0.099 |
| Hippocampus | **0.328** | **<0.001** | **-0.108** | **0.033** | -0.056 | 0.494 | **0.333** | **<0.001** |
| Entorhinal | **0.274** | **<0.001** | **-0.141** | **0.007** | **-0.171** | **0.042** | 0.134 | 0.161 |
| Mid temporal | **0.308** | **<0.001** | **-0.111** | **0.036** | -0.144 | 0.092 | **0.249** | **0.011** |
| Neurogranin | - | - | - | - | - | - | - | - |
| sTREM2 | 0.030 | 0.696 | 0.011 | 0.894 | 0.140 | 0.283 | 0.002 | 0.988 |
| YKL-40 | 0.223 | 0.120 | -0.243 | 0.101 | -0.102 | 0.709 | 0.244 | 0.593 |
| MEM | **0.435** | **<0.001** | **-0.111** | **0.008** | **-0.211** | **0.003** | 0.153 | 0.054 |
| EF | **0.432** | **<0.001** | **-0.126** | **0.003** | **-0.176** | **0.015** | 0.122 | 0.139 |
| LAN | **0.357** | **<0.001** | -0.070 | 0.120 | -0.147 | 0.051 | 0.101 | 0.235 |
| VS | **0.263** | **<0.001** | **-0.117** | **0.014** | -0.140 | 0.081 | 0.095 | 0.295 |

Significant effects (P <0.05) are shown in bold. Models included age, sex, education, *APOEε4* status and intracranial volume as covariates.

**Abbreviations:** MCI mild cognitive impairment; *APOEε4*, Apolipoprotein E4; Aβ, Amyloid-β; p-tau, Phosphorylated tau; t-tau, Total tau; NFL, Neurofilament light; sTREM2, Soluble triggering receptor on myeloid cells 2; MEM, Memory function; EF, Executive function; LAN, Language; VS, Visuospatial functioning.
