# Supplementary material for: P-tau and neurodegeneration mediate the effect of β-amyloid on cognition in non-demented elders
Source: Alzheimers Res Ther. 2021 Dec 15;13:200. doi: 10.1186/s13195-021-00943-z (PMC8675473; doi:10.1186/s13195-021-00943-z)
Supplement: Supplementary file 6 — Additional file 6. Effects of biomarkers on cognitive composite measures in CN participants. [file 13195_2021_943_MOESM6_ESM.docx]

**Additional file 6.** Effects of biomarkers on cognitive composite measures in CN participants.

|  | | **Baseline** | |  | **Longitudinal** | |  | **Biomarkers longitudinal** | |
| --- | --- | --- | --- | --- | --- | --- | --- | --- | --- |
|  |  | **β** | **P** |  | **β** | **P** |  | **β** | **P** |
| p-tau | MEM | 0.023 | 0.635 |  | -0.072 | 0.177 |  | -0.082 | 0.268 |
|  | EF | 0.019 | 0.695 |  | **-0.156** | **0.003** |  | **-0.175** | **0.018** |
|  | LAN | 0.070 | 0.169 |  | **-0.142** | **0.008** |  | **-0.225** | **0.003** |
|  | VS | 0.056 | 0.290 |  | -0.092 | 0.085 |  | **-0.193** | **0.008** |
| t-tau | MEM | 0.007 | 0.878 |  | **-0.125** | **0.019** |  | -0.013 | 0.859 |
|  | EF | -0.001 | 0.976 |  | **-0.127** | **0.016** |  | **-0.174** | **0.020** |
|  | LAN | 0.014 | 0.790 |  | -0.124 | 0.021 |  | -0.100 | 0.192 |
|  | VS | 0.090 | 0.095 |  | -0.075 | 0.165 |  | -0.137 | 0.065 |
| NFL | MEM | 0.075 | 0.125 |  | -0.053 | 0.329 |  | **-0.211** | **0.019** |
|  | EF | 0.067 | 0.175 |  | 0.004 | 0.943 |  | **-0.241** | **0.009** |
|  | LAN | 0.036 | 0.492 |  | -0.100 | 0.069 |  | **-0.309** | **0.001** |
|  | VS | 0.047 | 0.393 |  | 0.009 | 0.871 |  | **-0.251** | **0.007** |
| Whole brain | MEM | 0.122 | 0.200 |  | 0.199 | 0.058 |  | **0.155** | **0.049** |
|  | EF | **0.191** | **0.048** |  | **0.258** | **0.013** |  | **0.249** | **0.002** |
|  | LAN | 0.181 | 0.073 |  | 0.123 | 0.249 |  | 0.142 | 0.086 |
|  | VS | -0.056 | 0.594 |  | **0.317** | **0.003** |  | **0.237** | **0.003** |
| Hippocampus | MEM | 0.008 | 0.889 |  | **0.213** | **0.001** |  | **0.427** | **<0.001** |
|  | EF | 0.017 | 0.778 |  | **0.210** | **0.001** |  | **0.334** | **<0.001** |
|  | LAN | 0.038 | 0.555 |  | **0.149** | **0.026** |  | **0.235** | **0.006** |
|  | VS | -0.033 | 0.620 |  | **0.244** | **<0.001** |  | **0.311** | **<0.001** |
| Entorhinal | MEM | 0.016 | 0.775 |  | **0.156** | **0.013** |  | **0.227** | **0.006** |
|  | EF | 0.025 | 0.671 |  | 0.071 | 0.258 |  | **0.240** | **0.005** |
|  | LAN | 0.028 | 0.641 |  | **0.151** | **0.018** |  | **0.213** | **0.015** |
|  | VS | -0.011 | 0.864 |  | **0.165** | **0.009** |  | **0.265** | **0.002** |
| Mid temporal | MEM | **0.169** | **0.009** |  | **0.174** | **0.015** |  | **0.210** | **0.008** |
|  | EF | 0.084 | 0.202 |  | **0.215** | **0.003** |  | **0.235** | **0.004** |
|  | LAN | **0.175** | **0.010** |  | 0.076 | 0.299 |  | 0.124 | 0.141 |
|  | VS | 0.072 | 0.308 |  | **0.167** | **0.021** |  | **0.303** | **<0.001** |
| Neurogranin | MEM | **-0.163** | **0.003** |  | **-0.209** | **<0.001** |  | - | - |
|  | EF | -0.077 | 0.184 |  | **-0.127** | **0.024** |  | - | - |
|  | LAN | **-0.137** | **0.019** |  | **-0.179** | **0.002** |  | - | - |
|  | VS | **-0.120** | **0.039** |  | -0.040 | 0.496 |  | - | - |
| sTREM2 | MEM | 0.028 | 0.603 |  | **0.140** | **0.024** |  | -0.111 | 0.220 |
|  | EF | 0.055 | 0.312 |  | **0.141** | **0.024** |  | -0.034 | 0.713 |
|  | LAN | 0.031 | 0.587 |  | -0.088 | 0.152 |  | -0.061 | 0.517 |
|  | VS | 0.089 | 0.140 |  | -0.018 | 0.769 |  | 0.082 | 0.370 |
| YKL-40 | MEM | 0.056 | 0.694 |  | 0.035 | 0.815 |  | 0.070 | 0.632 |
|  | EF | 0.007 | 0.961 |  | 0.128 | 0.385 |  | -0.032 | 0.827 |
|  | LAN | -0.063 | 0.660 |  | 0.002 | 0.986 |  | 0.058 | 0.683 |
|  | VS | 0.123 | 0.349 |  | 0.049 | 0.742 |  | 0.139 | 0.335 |

­Significant effects (P <0.05) are shown in bold. Models included age, sex, education, *APOEε4* status and intracranial volume as covariates.

Abbreviations: CN, Normal controls; *APOEε4*, Apolipoprotein E4; p-tau, Phosphorylated tau; t-tau, Total tau; NFL, Neurofilament light; sTREM2, Soluble triggering receptor on myeloid cells 2; MEM, Memory function; EF, Executive function; LAN, Language; VS, Visuospatial functioning.
