# Supplementary material for: P-tau and neurodegeneration mediate the effect of β-amyloid on cognition in non-demented elders
Source: Alzheimers Res Ther. 2021 Dec 15;13:200. doi: 10.1186/s13195-021-00943-z (PMC8675473; doi:10.1186/s13195-021-00943-z)
Supplement: Supplementary file 7 — Additional file 7. Effects of biomarkers on cognitive composite measures in MCI participants. [file 13195_2021_943_MOESM7_ESM.docx]

**Additional file 7.** Effects of biomarkers on cognitive composite measures in MCI participants.

|  | | **Baseline** | |  | **Longitudinal** | |  | **Biomarkers longitudinal** | |
| --- | --- | --- | --- | --- | --- | --- | --- | --- | --- |
|  |  | **β** | **P** |  | **β** | **P** |  | **β** | **P** |
| p-tau | MEM | **-0.223** | **<0.001** |  | **-0.310** | **<0.001** |  | **-0.157** | **0.002** |
|  | EF | **-0.109** | **0.005** |  | **-0.277** | **<0.001** |  | -0.056 | 0.278 |
|  | LAN | **-0.101** | **0.009** |  | **-0.293** | **<0.001** |  | **-0.125** | **0.016** |
|  | VS | **-0.110** | **0.008** |  | **-0.169** | **<0.001** |  | -0.009 | 0.869 |
| t-tau | MEM | **-0.268** | **<0.001** |  | **-0.374** | **<0.001** |  | **-0.175** | **0.001** |
|  | EF | **-0.163** | **<0.001** |  | **-0.318** | **<0.001** |  | **-0.170** | **0.001** |
|  | LAN | **-0.172** | **<0.001** |  | **-0.341** | **<0.001** |  | **-0.143** | **0.006** |
|  | VS | **-0.140** | **<0.001** |  | **-0.184** | **<0.001** |  | -0.062 | 0.251 |
| NFL | MEM | **-0.168** | **<0.001** |  | **-0.221** | **<0.001** |  | **-0.170** | **0.013** |
|  | EF | **-0.158** | **<0.001** |  | **-0.190** | **<0.001** |  | **-0.221** | **0.002** |
|  | LAN | **-0.185** | **<0.001** |  | **-0.212** | **<0.001** |  | **-0.155** | **0.030** |
|  | VS | **-0.102** | **0.022** |  | -0.051 | 0.261 |  | **-0.172** | **0.018** |
| Whole brain | MEM | **0.536** | **<0.001** |  | **0.579** | **<0.001** |  | **0.357** | **<0.001** |
|  | EF | **0.499** | **<0.001** |  | **0.668** | **<0.001** |  | **0.327** | **<0.001** |
|  | LAN | **0.367** | **<0.001** |  | **0.640** | **<0.001** |  | **0.293** | **<0.001** |
|  | VS | **0.222** | **0.004** |  | **0.397** | **<0.001** |  | **0.245** | **<0.001** |
| Hippocampus | MEM | **0.428** | **<0.001** |  | **0.409** | **<0.001** |  | **0.557** | **<0.001** |
|  | EF | **0.233** | **<0.001** |  | **0.382** | **<0.001** |  | **0.476** | **<0.001** |
|  | LAN | **0.266** | **<0.001** |  | **0.375** | **<0.001** |  | **0.467** | **<0.001** |
|  | VS | **0.161** | **0.002** |  | **0.206** | **<0.001** |  | **0.308** | **<0.001** |
| Entorhinal | MEM | **0.353** | **<0.001** |  | **0.294** | **<0.001** |  | **0.439** | **<0.001** |
|  | EF | **0.193** | **<0.001** |  | **0.260** | **<0.001** |  | **0.361** | **<0.001** |
|  | LAN | **0.210** | **<0.001** |  | **0.364** | **<0.001** |  | **0.422** | **<0.001** |
|  | VS | 0.081 | 0.090 |  | **0.177** | **<0.001** |  | **0.247** | **<0.001** |
| Mid temporal | MEM | **0.383** | **<0.001** |  | **0.442** | **<0.001** |  | **0.539** | **<0.001** |
|  | EF | **0.319** | **<0.001** |  | **0.498** | **<0.001** |  | **0.501** | **<0.001** |
|  | LAN | **0.302** | **<0.001** |  | **0.493** | **<0.001** |  | **0.556** | **<0.001** |
|  | VS | **0.146** | **0.005** |  | **0.286** | **<0.001** |  | **0.389** | **<0.001** |
| Neurogranin | MEM | -0.060 | 0.428 |  | **-0.148** | **0.041** |  | - | - |
|  | EF | -0.012 | 0.871 |  | -0.058 | 0.429 |  | - | - |
|  | LAN | -0.058 | 0.439 |  | -0.105 | 0.156 |  | - | - |
|  | VS | -0.050 | 0.505 |  | 0.023 | 0.763 |  | - | - |
| sTREM2 | MEM | -0.064 | 0.122 |  | 0.007 | 0.861 |  | -0.053 | 0.404 |
|  | EF | -0.047 | 0.262 |  | -0.054 | 0.214 |  | -0.026 | 0.690 |
|  | LAN | -0.011 | 0.791 |  | -0.044 | 0.313 |  | -0.088 | 0.183 |
|  | VS | -0.026 | 0.555 |  | -0.043 | 0.350 |  | 0.035 | 0.595 |
| YKL-40 | MEM | -0.066 | 0.595 |  | 0.017 | 0.889 |  | 0.148 | 0.204 |
|  | EF | -0.070 | 0.582 |  | 0.191 | 0.097 |  | 0.056 | 0.616 |
|  | LAN | **-0.258** | **0.035** |  | 0.139 | 0.256 |  | 0.080 | 0.493 |
|  | VS | -0.056 | 0.661 |  | 0.165 | 0.171 |  | 0.116 | 0.318 |

­Significant effects (P <0.05) are shown in bold. Models included age, sex, education, *APOEε4* status and intracranial volume as covariates.

Abbreviations: MCI mild cognitive impairment; *APOEε4*, Apolipoprotein E4; p-tau, Phosphorylated tau; t-tau, Total tau; NFL, Neurofilament light; sTREM2, Soluble triggering receptor on myeloid cells 2; MEM, Memory function; EF, Executive function; LAN, Language; VS, Visuospatial functioning.
