# Supplementary material for: P-tau and neurodegeneration mediate the effect of β-amyloid on cognition in non-demented elders
Source: Alzheimers Res Ther. 2021 Dec 15;13:200. doi: 10.1186/s13195-021-00943-z (PMC8675473; doi:10.1186/s13195-021-00943-z)
Supplement: Supplementary file 9 — Additional file 9. Mediation analyses of Aβ and cognitive measurements with biomarkers as mediators in A+CN participants. [file 13195_2021_943_MOESM9_ESM.docx]

**Additional file 9.** Mediation analyses of Aβ and cognitive measurements with biomarkers as mediators in A+CN participants.

| **A+CN** |  | **a** | **P** | **b** | **P** | **c** | **P** | **c’** | **P** | **Proportion (%)** | | **P** |
| --- | --- | --- | --- | --- | --- | --- | --- | --- | --- | --- | --- | --- |
| **Baseline** |  |  |  |  |  |  |  |  |  | |  |  |
| p-tau | MEM | **-0.23** | **0.01** | 0.14 | 0.07 | 0.06 | 0.41 | 0.10 | 0.21 | | 27.25 | 0.04 |
|  | EF | **-0.23** | **0.01** | 0.09 | 0.26 | -0.01 | 0.93 | 0.01 | 0.88 | | 6.06 | 0.27 |
|  | LAN | **-0.23** | **0.01** | 0.09 | 0.24 | -0.09 | 0.27 | -0.08 | 0.38 | | 11.81 | 0.33 |
|  | VS | **-0.23** | **0.01** | 0.06 | 0.49 | 0.01 | 0.92 | 0.02 | 0.80 | | 3.18 | 0.43 |
| t-tau | MEM | **-0.17** | **0.04** | 0.14 | 0.07 | 0.06 | 0.41 | 0.09 | 0.25 | | 18.11 | 0.09 |
|  | EF | **-0.17** | **0.04** | 0.14 | 0.07 | 0.06 | 0.41 | 0.09 | 0.25 | | 18.11 | 0.09 |
|  | LAN | **-0.17** | **0.04** | 0.04 | 0.59 | -0.09 | 0.27 | -0.09 | 0.30 | | 2.17 | 0.78 |
|  | VS | **-0.17** | **0.04** | **0.17** | **0.04** | 0.01 | 0.92 | 0.04 | 0.66 | | 6.84 | 0.07 |
| NFL | MEM | -0.01 | 0.87 | 0.01 | 0.85 | 0.08 | 0.30 | 0.08 | 0.30 | | 0.08 | 0.91 |
|  | EF | -0.01 | 0.87 | 0.03 | 0.75 | -0.01 | 0.93 | -0.01 | 0.93 | | 0.68 | 0.95 |
|  | LAN | -0.01 | 0.87 | 0.02 | 0.85 | -0.09 | 0.29 | -0.09 | 0.29 | | 0.18 | 0.98 |
|  | VS | -0.01 | 0.87 | **0.19** | **0.03** | 0.01 | 0.91 | 0.01 | 0.88 | | 0.26 | 0.92 |
| Whole brain | MEM | 0.01 | 0.87 | 0.27 | 0.07 | 0.01 | 0.96 | -0.01 | 0.98 | | 6.73 | 0.78 |
|  | EF | 0.01 | 0.87 | 0.20 | 0.20 | -0.03 | 0.77 | -0.03 | 0.75 | | 2.57 | 0.86 |
|  | LAN | 0.01 | 0.87 | 0.23 | 0.16 | -0.11 | 0.22 | -0.12 | 0.20 | | 0.06 | 0.92 |
|  | VS | 0.01 | 0.87 | 0.08 | 0.63 | -0.01 | 0.95 | -0.01 | 0.94 | | 0.41 | 0.88 |
| Hippocampus | MEM | 0.01 | 0.88 | 0.01 | 0.98 | 0.01 | 0.96 | 0.0.1 | 0.96 | | 0.43 | 0.99 |
|  | EF | 0.01 | 0.88 | -0.09 | 0.38 | -0.03 | 0.77 | -0.02 | 0.80 | | 0.31 | 0.92 |
|  | LAN | 0.01 | 0.88 | 0.01 | 0.94 | -0.11 | 0.22 | -0.11 | 0.23 | | 0.18 | 0.94 |
|  | VS | 0.01 | 0.88 | 0.08 | 0.41 | -0.01 | 0.95 | -0.01 | 0.95 | | 0.21 | 0.90 |
| Entorhinal | MEM | 0.07 | 0.42 | 0.01 | 0.89 | 0.01 | 0.96 | 0.01 | 0.98 | | 0.34 | 0.90 |
|  | EF | 0.07 | 0.42 | -0.05 | 0.64 | -0.03 | 0.77 | -0.02 | 0.81 | | 0.37 | 0.78 |
|  | LAN | 0.07 | 0.42 | -0.03 | 0.73 | -0.11 | 0.22 | -0.11 | 0.24 | | 0.06 | 0.91 |
|  | VS | 0.07 | 0.42 | -0.11 | 0.30 | -0.01 | 0.95 | 0.01 | 0.98 | | 0.93 | 0.60 |
| Mid temporal | MEM | 0.02 | 0.78 | **0.23** | **0.02** | 0.01 | 0.96 | -0.01 | 0.97 | | 5.07 | 0.70 |
|  | EF | 0.02 | 0.78 | 0.13 | 0.19 | -0.03 | 0.77 | -0.03 | 0.75 | | 1.44 | 0.76 |
|  | LAN | 0.02 | 0.78 | 0.16 | 0.13 | -0.11 | 0.22 | -0.12 | 0.20 | | 0.71 | 0.85 |
|  | VS | 0.02 | 0.78 | 0.07 | 0.51 | -0.01 | 0.95 | -0.01 | 0.94 | | 0.64 | 0.78 |
| Neurogranin | MEM | -0.16 | 0.37 | -0.27 | 0.06 | **0.34** | **0.02** | **0.30** | **0.03** | | 7.88 | 0.39 |
|  | EF | -0.16 | 0.37 | 0.10 | 0.50 | -0.08 | 0.59 | -0.07 | 0.66 | | 1.92 | 0.69 |
|  | LAN | -0.16 | 0.37 | -0.21 | 0.17 | 0.15 | 0.34 | 0.12 | 0.45 | | 11.31 | 0.42 |
|  | VS | -0.16 | 0.37 | **-0.44** | **0.00** | 0.08 | 0.61 | 0.01 | 0.93 | | 33.06 | 0.33 |
| sTREM2 | MEM | 0.09 | 0.34 | 0.04 | 0.70 | 0.10 | 0.29 | 0.09 | 0.30 | | 0.49 | 0.87 |
|  | EF | 0.09 | 0.34 | 0.14 | 0.12 | -0.07 | 0.46 | -0.08 | 0.38 | | 5.79 | 0.38 |
|  | LAN | 0.09 | 0.34 | -0.06 | 0.54 | -0.12 | 0.20 | -0.12 | 0.22 | | 1.68 | 0.69 |
|  | VS | 0.09 | 0.34 | 0.05 | 0.61 | 0.01 | 0.89 | 0.01 | 0.93 | | 0.18 | 0.75 |
| YKL-40 | MEM | -0.22 | 0.30 | 0.18 | 0.58 | 0.16 | 0.56 | 0.21 | 0.45 | | 2.86 | 0.59 |
|  | EF | -0.22 | 0.30 | 0.12 | 0.68 | 0.22 | 0.32 | 0.21 | 0.38 | | 1.14 | 0.94 |
|  | LAN | -0.22 | 0.30 | 0.13 | 0.63 | -0.06 | 0.79 | -0.03 | 0.89 | | 2.98 | 0.77 |
|  | VS | -0.22 | 0.30 | 0.28 | 0.38 | -0.03 | 0.90 | 0.03 | 0.90 | | 2.83 | 0.56 |
| **Longitudinal** |  |  |  |  |  |  |  |  |  | |  |  |
| p-tau | MEM | -0.03 | 0.81 | -0.09 | 0.46 | 0.14 | 0.27 | 0.17 | 0.16 | | 1.00 | 0.80 |
|  | EF | -0.03 | 0.81 | -0.17 | 0.18 | 0.03 | 0.84 | 0.04 | 0.76 | | 2.67 | 0.84 |
|  | LAN | -0.03 | 0.81 | -0.13 | 0.31 | 0.09 | 0.49 | 0.06 | 0.64 | | 0.85 | 0.97 |
|  | VS | -0.03 | 0.81 | -0.16 | 0.19 | 0.19 | 0.12 | 0.19 | 0.11 | | 1.15 | 0.82 |
| t-tau | MEM | -0.08 | 0.51 | 0.06 | 0.63 | 0.14 | 0.27 | 0.18 | 0.16 | | 1.56 | 0.72 |
|  | EF | -0.08 | 0.51 | -0.13 | 0.30 | 0.03 | 0.84 | 0.02 | 0.84 | | 2.26 | 0.66 |
|  | LAN | -0.08 | 0.51 | 0.06 | 0.66 | 0.09 | 0.49 | 0.06 | 0.62 | | 0.36 | 0.84 |
|  | VS | -0.08 | 0.51 | -0.15 | 0.20 | 0.19 | 0.12 | 0.17 | 0.14 | | 3.38 | 0.64 |
| NFL | MEM | -0.04 | 0.76 | -0.21 | 0.17 | 0.10 | 0.49 | 0.16 | 0.26 | | 3.04 | 0.86 |
|  | EF | -0.04 | 0.76 | -0.20 | 0.22 | 0.04 | 0.81 | 0.04 | 0.76 | | 2.83 | 0.83 |
|  | LAN | -0.04 | 0.76 | -0.17 | 0.29 | 0.13 | 0.41 | 0.09 | 0.52 | | 2.28 | 0.81 |
|  | VS | -0.04 | 0.76 | -0.10 | 0.53 | 0.15 | 0.31 | 0.15 | 0.28 | | 0.78 | 0.96 |
| Whole brain | MEM | **0.33** | **0.02** | **0.38** | **0.00** | 0.15 | 0.28 | 0.10 | 0.44 | | 51.72 | 0.03 |
|  | EF | **0.33** | **0.02** | **0.30** | **0.03** | 0.03 | 0.86 | -0.04 | 0.75 | | 50.14 | 0.03 |
|  | LAN | **0.33** | **0.02** | 0.25 | 0.07 | 0.10 | 0.51 | -0.02 | 0.91 | | 34.27 | 0.10 |
|  | VS | **0.33** | **0.02** | **0.28** | **0.04** | 0.10 | 0.49 | 0.04 | 0.78 | | 0.44 | 0.04 |
| Hippocampus | MEM | **0.43** | **0.00** | **0.48** | **0.00** | 0.15 | 0.28 | -0.03 | 0.84 | | 10.65 | 0.00 |
|  | EF | **0.43** | **0.00** | 0.23 | 0.10 | 0.03 | 0.86 | -0.08 | 0.61 | | 0.29 | 0.11 |
|  | LAN | **0.43** | **0.00** | 0.21 | 0.13 | 0.10 | 0.51 | -0.05 | 0.73 | | 35.93 | 0.12 |
|  | VS | **0.43** | **0.00** | 0.24 | 0.07 | 0.10 | 0.49 | 0.01 | 0.99 | | 47.67 | 0.09 |
| Entorhinal | MEM | 0.10 | 0.45 | **0.33** | **0.02** | 0.15 | 0.28 | 0.16 | 0.22 | | 13.59 | 0.44 |
|  | EF | 0.10 | 0.45 | 0.25 | 0.08 | 0.03 | 0.86 | 0.01 | 0.94 | | 0.06 | 0.48 |
|  | LAN | 0.10 | 0.45 | 0.27 | 0.06 | 0.10 | 0.51 | 0.02 | 0.90 | | 8.94 | 0.47 |
|  | VS | 0.10 | 0.45 | 0.24 | 0.08 | 0.10 | 0.49 | 0.08 | 0.54 | | 10.00 | 0.51 |
| Mid temporal | MEM | 0.21 | 0.13 | **0.31** | **0.02** | 0.15 | 0.28 | 0.14 | 0.31 | | 24.28 | 0.16 |
|  | EF | 0.21 | 0.13 | 0.13 | 0.37 | 0.03 | 0.86 | 0.02 | 0.91 | | 4.36 | 0.50 |
|  | LAN | 0.21 | 0.13 | -0.06 | 0.67 | 0.10 | 0.51 | 0.07 | 0.61 | | 4.39 | 0.63 |
|  | VS | 0.21 | 0.13 | 0.14 | 0.31 | 0.10 | 0.49 | 0.09 | 0.53 | | 7.64 | 0.48 |
| sTREM2 | MEM | 0.11 | 0.49 | -0.19 | 0.23 | 0.04 | 0.78 | 0.10 | 0.50 | | 1.63 | 0.61 |
|  | EF | 0.11 | 0.49 | -0.24 | 0.13 | -0.02 | 0.89 | -0.01 | 0.93 | | 5.53 | 0.57 |
|  | LAN | 0.11 | 0.49 | -0.25 | 0.10 | 0.05 | 0.73 | 0.04 | 0.78 | | 2.17 | 0.54 |
|  | VS | 0.11 | 0.49 | 0.06 | 0.70 | 0.16 | 0.30 | 0.15 | 0.33 | | 1.32 | 0.90 |
| YKL-40 | MEM | 0.06 | 0.79 | 0.34 | 0.26 | 0.40 | 0.14 | 0.38 | 0.15 | | 2.26 | 0.82 |
|  | EF | 0.06 | 0.79 | 0.41 | 0.16 | 0.36 | 0.18 | 0.34 | 0.19 | | 5.36 | 0.81 |
|  | LAN | 0.06 | 0.79 | 0.40 | 0.16 | 0.46 | 0.08 | 0.39 | 0.12 | | 4.49 | 0.85 |
|  | VS | 0.06 | 0.79 | 0.29 | 0.31 | 0.45 | 0.09 | 0.42 | 0.10 | | 1.42 | 0.87 |

Significant effects (P <0.05) are shown in bold. Models included age, sex, education, *APOEε4* status and intracranial volume as covariates.

Abbreviations: CN, Normal controls; *APOEε4*, Apolipoprotein E4; p-tau, Phosphorylated tau; t-tau, Total tau; NFL, Neurofilament light; sTREM2, Soluble triggering receptor on myeloid cells 2; MEM, Memory function; EF, Executive function; LAN, Language; VS, Visuospatial functioning.
