# Supplementary material for: P-tau and neurodegeneration mediate the effect of β-amyloid on cognition in non-demented elders
Source: Alzheimers Res Ther. 2021 Dec 15;13:200. doi: 10.1186/s13195-021-00943-z (PMC8675473; doi:10.1186/s13195-021-00943-z)
Supplement: Supplementary file 10 — Additional file 10. Mediation analyses of Aβ and cognitive measurements with biomarkers as mediators in A-MCI participants. [file 13195_2021_943_MOESM10_ESM.docx]

**Additional file 10.** Mediation analyses of Aβ and cognitive measurements with biomarkers as mediators in A-MCI participants.

| **A-MCI** |  | **a** | **P** | **b** | **P** | **c** | **P** | **c’** | **P** | **Proportion (%)** | | **P** |
| --- | --- | --- | --- | --- | --- | --- | --- | --- | --- | --- | --- | --- |
| **Baseline** |  |  |  |  |  |  |  |  |  | |  |  |
| p-tau | MEM | 0.06 | 0.40 | -0.12 | 0.05 | -0.05 | 0.44 | -0.04 | 0.51 | | 5.78 | 0.44 |
|  | EF | 0.06 | 0.40 | -0.07 | 0.25 | 0.03 | 0.65 | 0.03 | 0.60 | | 1.62 | 0.56 |
|  | LAN | 0.06 | 0.40 | -0.07 | 0.28 | 0.04 | 0.57 | 0.04 | 0.52 | | 1.53 | 0.61 |
|  | VS | 0.06 | 0.40 | -0.02 | 0.73 | -0.04 | 0.58 | -0.04 | 0.60 | | 0.31 | 0.76 |
| t-tau | MEM | **0.28** | **0.00** | -0.05 | 0.40 | -0.05 | 0.44 | -0.03 | 0.59 | | 12.31 | 0.49 |
|  | EF | **0.28** | **0.00** | 0.05 | 0.41 | 0.03 | 0.65 | 0.01 | 0.82 | | 13.75 | 0.44 |
|  | LAN | **0.28** | **0.00** | -0.02 | 0.75 | 0.04 | 0.57 | 0.05 | 0.49 | | 8.38 | 0.60 |
|  | VS | **0.28** | **0.00** | -0.08 | 0.25 | -0.04 | 0.58 | -0.02 | 0.82 | | 17.15 | 0.32 |
| NFL | MEM | -0.01 | 0.86 | **-0.14** | **0.04** | -0.05 | 0.44 | -0.05 | 0.42 | | 0.20 | 0.89 |
|  | EF | -0.01 | 0.86 | **-0.16** | **0.02** | 0.03 | 0.64 | 0.03 | 0.65 | | 2.74 | 0.84 |
|  | LAN | -0.01 | 0.86 | -0.10 | 0.17 | 0.03 | 0.60 | 0.03 | 0.61 | | 0.01 | 0.91 |
|  | VS | -0.01 | 0.86 | -0.11 | 0.16 | -0.04 | 0.56 | -0.04 | 0.55 | | 0.33 | 0.84 |
| Whole brain | MEM | -0.07 | 0.26 | **0.47** | **0.00** | 0.02 | 0.73 | 0.01 | 0.97 | | 20.83 | 0.30 |
|  | EF | -0.07 | 0.26 | **0.47** | **0.00** | 0.01 | 0.84 | 0.00 | 0.97 | | 25.10 | 0.31 |
|  | LAN | -0.07 | 0.26 | **0.42** | **0.00** | 0.06 | 0.42 | 0.06 | 0.41 | | 0.70 | 0.27 |
|  | VS | -0.07 | 0.26 | -0.01 | 0.97 | 0.01 | 0.85 | 0.02 | 0.74 | | 1.74 | 0.94 |
| Hippocampus | MEM | 0.04 | 0.54 | **0.20** | **0.01** | 0.02 | 0.73 | -0.01 | 0.98 | | 5.27 | 0.54 |
|  | EF | 0.04 | 0.54 | 0.15 | 0.07 | 0.01 | 0.84 | 0.01 | 0.96 | | 1.17 | 0.60 |
|  | LAN | 0.04 | 0.54 | **0.18** | **0.03** | 0.06 | 0.42 | 0.06 | 0.43 | | 4.15 | 0.52 |
|  | VS | 0.04 | 0.54 | -0.02 | 0.86 | 0.01 | 0.85 | 0.03 | 0.73 | | 0.09 | 0.90 |
| Entorhinal | MEM | 0.11 | 0.13 | **0.27** | **0.00** | 0.02 | 0.73 | -0.03 | 0.64 | | 16.08 | 0.16 |
|  | EF | 0.11 | 0.13 | 0.14 | 0.06 | 0.01 | 0.84 | -0.01 | 0.91 | | 5.38 | 0.19 |
|  | LAN | 0.11 | 0.13 | 0.15 | 0.05 | 0.06 | 0.42 | 0.05 | 0.53 | | 12.20 | 0.19 |
|  | VS | 0.11 | 0.13 | 0.07 | 0.35 | 0.01 | 0.85 | 0.01 | 0.86 | | 1.69 | 0.50 |
| Mid temporal | MEM | -0.10 | 0.15 | **0.23** | **0.00** | 0.02 | 0.73 | 0.02 | 0.74 | | 0.97 | 0.18 |
|  | EF | -0.10 | 0.15 | 0.14 | 0.07 | 0.01 | 0.84 | 0.02 | 0.77 | | 1.99 | 0.18 |
|  | LAN | -0.10 | 0.15 | 0.15 | 0.08 | 0.06 | 0.42 | 0.07 | 0.30 | | 10.39 | 0.26 |
|  | VS | -0.10 | 0.15 | 0.02 | 0.84 | 0.01 | 0.85 | 0.03 | 0.93 | | 0.05 | 0.90 |
| Neurogranin | MEM | 0.20 | 0.16 | **0.33** | **0.02** | 0.16 | 0.24 | 0.10 | 0.46 | | 28.47 | 0.17 |
|  | EF | 0.20 | 0.16 | **0.30** | **0.04** | -0.11 | 0.43 | -0.18 | 0.20 | | 19.88 | 0.20 |
|  | LAN | 0.20 | 0.16 | 0.23 | 0.14 | -0.09 | 0.54 | -0.14 | 0.34 | | 13.06 | 0.24 |
|  | VS | 0.20 | 0.16 | 0.23 | 0.14 | -0.09 | 0.54 | -0.14 | 0.34 | | 13.06 | 0.24 |
| sTREM2 | MEM | **0.15** | **0.04** | 0.02 | 0.76 | -0.07 | 0.28 | -0.08 | 0.26 | | 2.36 | 0.68 |
|  | EF | **0.15** | **0.04** | -0.01 | 0.91 | -0.01 | 0.94 | -0.01 | 0.95 | | 0.42 | 0.94 |
|  | LAN | **0.15** | **0.04** | 0.08 | 0.25 | -0.01 | 0.95 | -0.02 | 0.81 | | 1.62 | 0.32 |
|  | VS | **0.15** | **0.04** | 0.14 | 0.08 | -0.11 | 0.15 | -0.13 | 0.08 | | 16.04 | 0.06 |
| YKL-40 | MEM | 0.29 | 0.26 | -0.04 | 0.89 | **0.56** | **0.02** | **0.64** | **0.01** | | 11.42 | 0.45 |
|  | EF | 0.29 | 0.26 | 0.25 | 0.42 | 0.06 | 0.84 | -0.02 | 0.95 | | 5.27 | 0.56 |
|  | LAN | 0.29 | 0.26 | 0.11 | 0.75 | **0.58** | **0.03** | **0.61** | **0.03** | | 3.16 | 0.69 |
|  | VS | 0.29 | 0.26 | -0.02 | 0.95 | -0.38 | 0.17 | -0.42 | 0.17 | | 2.23 | 0.77 |
| **Longitudinal** |  |  |  |  |  |  |  |  |  | |  |  |
| p-tau | MEM | -0.04 | 0.65 | 0.04 | 0.68 | 0.01 | 0.89 | 0.01 | 0.89 | | 0.37 | 0.86 |
|  | EF | -0.04 | 0.65 | 0.04 | 0.69 | 0.05 | 0.59 | 0.05 | 0.59 | | 0.15 | 0.89 |
|  | LAN | -0.04 | 0.65 | 0.01 | 0.98 | -0.06 | 0.52 | -0.05 | 0.55 | | 0.36 | 0.95 |
|  | VS | -0.04 | 0.65 | -0.02 | 0.87 | -0.22 | 0.03 | **-0.22** | **0.03** | | 0.07 | 0.95 |
| t-tau | MEM | 0.07 | 0.48 | 0.06 | 0.51 | 0.01 | 0.89 | 0.01 | 0.94 | | 0.35 | 0.72 |
|  | EF | 0.07 | 0.48 | 0.02 | 0.84 | 0.05 | 0.59 | 0.05 | 0.60 | | 0.19 | 0.88 |
|  | LAN | 0.07 | 0.48 | 0.01 | 0.99 | -0.06 | 0.55 | -0.05 | 0.54 | | -0.01 | 0.93 |
|  | VS | 0.07 | 0.48 | 0.02 | 0.82 | **-0.22** | **0.03** | **-0.22** | **0.02** | | 0.24 | 0.92 |
| NFL | MEM | -0.15 | 0.10 | **-0.37** | **0.00** | 0.02 | 0.83 | -0.04 | 0.67 | | 20.56 | 0.05 |
|  | EF | -0.15 | 0.10 | **-0.32** | **0.01** | 0.01 | 0.95 | -0.05 | 0.66 | | 10.33 | 0.07 |
|  | LAN | -0.15 | 0.10 | **-0.27** | **0.02** | -0.06 | 0.56 | -0.10 | 0.29 | | 20.08 | 0.11 |
|  | VS | -0.15 | 0.10 | -0.01 | 0.92 | **-0.31** | **0.00** | **-0.32** | **0.00** | | 3.13 | 0.57 |
| Whole brain | MEM | 0.02 | 0.86 | **0.26** | **0.01** | 0.05 | 0.63 | 0.04 | 0.70 | | 5.91 | 0.83 |
|  | EF | 0.02 | 0.86 | **0.22** | **0.04** | 0.05 | 0.64 | 0.04 | 0.67 | | 3.66 | 0.86 |
|  | LAN | 0.02 | 0.86 | 0.15 | 0.13 | -0.01 | 0.92 | -0.02 | 0.85 | | 0.48 | 0.84 |
|  | VS | 0.02 | 0.86 | -0.01 | 0.91 | -0.18 | 0.10 | -0.19 | 0.09 | | 0.03 | 0.97 |
| Hippocampus | MEM | 0.11 | 0.26 | **0.48** | **0.00** | 0.05 | 0.63 | -0.02 | 0.80 | | 35.20 | 0.24 |
|  | EF | 0.11 | 0.26 | **0.28** | **0.01** | 0.05 | 0.64 | 0.01 | 0.95 | | 14.88 | 0.30 |
|  | LAN | 0.11 | 0.26 | **0.28** | **0.01** | -0.01 | 0.92 | -0.06 | 0.56 | | 6.74 | 0.28 |
|  | VS | 0.11 | 0.26 | 0.01 | 0.91 | -0.18 | 0.10 | -0.20 | 0.08 | | 1.06 | 0.74 |
| Entorhinal | MEM | -0.06 | 0.56 | **0.37** | **0.00** | 0.05 | 0.63 | 0.05 | 0.64 | | 6.50 | 0.54 |
|  | EF | -0.06 | 0.56 | 0.14 | 0.22 | 0.05 | 0.64 | 0.05 | 0.67 | | 0.24 | 0.64 |
|  | LAN | -0.06 | 0.56 | 0.18 | 0.11 | -0.01 | 0.92 | -0.02 | 0.87 | | 2.80 | 0.64 |
|  | VS | -0.06 | 0.56 | 0.13 | 0.27 | -0.18 | 0.10 | -0.19 | 0.09 | | 2.71 | 0.58 |
| Mid temporal | MEM | -0.05 | 0.65 | **0.28** | **0.01** | 0.05 | 0.63 | 0.04 | 0.67 | | 1.05 | 0.64 |
|  | EF | -0.05 | 0.65 | **0.28** | **0.01** | 0.05 | 0.64 | 0.05 | 0.64 | | 0.73 | 0.61 |
|  | LAN | -0.05 | 0.65 | 0.17 | 0.11 | -0.01 | 0.92 | -0.02 | 0.87 | | 3.36 | 0.76 |
|  | VS | -0.05 | 0.65 | **0.23** | **0.04** | -0.18 | 0.10 | -0.19 | 0.09 | | 3.19 | 0.69 |
| sTREM2 | MEM | 0.09 | 0.44 | 0.03 | 0.80 | 0.02 | 0.83 | 0.01 | 0.98 | | 0.75 | 0.97 |
|  | EF | 0.09 | 0.44 | -0.08 | 0.47 | 0.03 | 0.77 | 0.03 | 0.78 | | 0.12 | 0.76 |
|  | LAN | 0.09 | 0.44 | -0.20 | 0.05 | -0.02 | 0.84 | -0.01 | 0.93 | | 7.14 | 0.49 |
|  | VS | 0.09 | 0.44 | 0.02 | 0.87 | **-0.26** | **0.02** | **-0.26** | **0.02** | | 0.36 | 0.84 |
| YKL-40 | MEM | 0.06 | 0.79 | 0.06 | 0.87 | -0.03 | 0.92 | -0.03 | 0.92 | | 0.36 | 0.84 |
|  | EF | 0.06 | 0.79 | 0.36 | 0.37 | 0.04 | 0.89 | 0.02 | 0.95 | | 0.36 | 0.84 |
|  | LAN | 0.06 | 0.79 | 0.51 | 0.12 | -0.17 | 0.53 | -0.20 | 0.42 | | 4.82 | 0.73 |
|  | VS | 0.06 | 0.79 | 0.10 | 0.80 | -0.36 | 0.22 | -0.36 | 0.23 | | 0.15 | 0.96 |

Significant effects (P <0.05) are shown in bold. Models included age, sex, education, *APOEε4* status and intracranial volume as covariates.

Abbreviations: MCI mild cognitive impairment; *APOEε4*, Apolipoprotein E4; p-tau, Phosphorylated tau; t-tau, Total tau; NFL, Neurofilament light; sTREM2, Soluble triggering receptor on myeloid cells 2; MEM, Memory function; EF, Executive function; LAN, Language; VS, Visuospatial functioning.
