# Supplementary material for: P-tau and neurodegeneration mediate the effect of β-amyloid on cognition in non-demented elders
Source: Alzheimers Res Ther. 2021 Dec 15;13:200. doi: 10.1186/s13195-021-00943-z (PMC8675473; doi:10.1186/s13195-021-00943-z)
Supplement: Supplementary file 11 — Additional file 11. Mediation analyses of Aβ and cognitive measurements with biomarkers as mediators in A+MCI participants. [file 13195_2021_943_MOESM11_ESM.docx]

**Additional file 11.** Mediation analyses of Aβ and cognitive measurements with biomarkers as mediators in A+MCI participants.

| **A+MCI** |  | **a** | **P** | **b** | **P** | **c** | **P** | **c’** | **P** | **Proportion (%)** | | **P** |
| --- | --- | --- | --- | --- | --- | --- | --- | --- | --- | --- | --- | --- |
| **Baseline** |  |  |  |  |  |  |  |  |  | |  |  |
| p-tau | MEM | **-0.24** | **<0.001** | **-0.14** | **<0.001** | **0.17** | **<0.001** | **0.14** | **0.01** | | **15.64** | **0.03** |
|  | EF | **-0.24** | **<0.001** | -0.02 | 0.70 | **0.10** | **0.04** | 0.10 | 0.05 | | 1.41 | 0.88 |
|  | LAN | **-0.24** | **<0.001** | -0.06 | 0.21 | **0.12** | **0.02** | **0.11** | **0.04** | | 6.09 | 0.55 |
|  | VS | **-0.24** | **<0.001** | -0.10 | 0.05 | 0.06 | 0.24 | 0.04 | 0.46 | | 25.40 | 0.06 |
| t-tau | MEM | **-0.22** | **<0.001** | **-0.24** | **<0.001** | **0.17** | **0.001** | **0.12** | **0.02** | | **18.20** | **0.03** |
|  | EF | **-0.22** | **<0.001** | **-0.14** | **0.01** | **0.10** | **0.04** | 0.08 | 0.14 | | 25.32 | 0.02 |
|  | LAN | **-0.22** | **<0.001** | **-0.17** | **<0.001** | **0.12** | **0.02** | 0.08 | 0.10 | | 27.58 | <0.001 |
|  | VS | **-0.22** | **<0.001** | **-0.13** | **0.01** | 0.06 | 0.24 | 0.04 | 0.51 | | 32.49 | 0.02 |
| NFL | MEM | -0.04 | 0.39 | **-0.17** | **0.01** | **0.18** | **<0.001** | **0.18** | **<0.001** | | 3.78 | 0.40 |
|  | EF | -0.04 | 0.39 | **-0.14** | **0.01** | 0.10 | 0.06 | 0.09 | 0.07 | | 4.56 | 0.43 |
|  | LAN | -0.04 | 0.39 | **-0.21** | **<0.001** | **0.14** | **0.01** | **0.13** | **0.01** | | 5.83 | 0.40 |
|  | VS | -0.04 | 0.39 | -0.09 | 0.12 | 0.04 | 0.48 | 0.03 | 0.52 | | 2.47 | 0.46 |
| Whole brain | MEM | 0.05 | 0.34 | **0.58** | **<0.001** | **0.22** | **<0.001** | **0.19** | **<0.001** | | 11.73 | 0.32 |
|  | EF | 0.05 | 0.34 | **0.47** | **<0.001** | 0.10 | 0.08 | 0.08 | 0.16 | | 22.71 | 0.34 |
|  | LAN | 0.05 | 0.34 | **0.32** | **<0.001** | 0.10 | 0.08 | 0.08 | 0.13 | | 14.57 | 0.34 |
|  | VS | 0.05 | 0.34 | **0.28** | **0.01** | 0.07 | 0.26 | 0.05 | 0.36 | | 11.52 | 0.31 |
| Hippocampus | MEM | **0.17** | **0.01** | **0.51** | **<0.001** | **0.22** | **<0.001** | **0.14** | **0.01** | | 36.55 | <0.001 |
|  | EF | **0.17** | **0.01** | **0.24** | **<0.001** | 0.10 | 0.08 | 0.06 | 0.27 | | 33.48 | 0.01 |
|  | LAN | **0.17** | **0.01** | **0.28** | **<0.001** | 0.10 | 0.08 | 0.05 | 0.34 | | 0.43 | 0.01 |
|  | VS | **0.17** | **0.01** | **0.22** | **<0.001** | 0.07 | 0.26 | 0.03 | 0.61 | | 43.60 | <0.001 |
| Entorhinal | MEM | 0.09 | 0.11 | **0.39** | **<0.001** | **0.22** | **<0.001** | **0.18** | **<0.001** | | 15.20 | 0.10 |
|  | EF | 0.09 | 0.11 | **0.19** | **<0.001** | 0.10 | 0.08 | 0.08 | 0.14 | | 14.90 | 0.11 |
|  | LAN | 0.09 | 0.11 | **0.22** | **<0.001** | 0.10 | 0.08 | 0.08 | 0.15 | | 16.70 | 0.15 |
|  | VS | 0.09 | 0.11 | 0.07 | 0.22 | 0.07 | 0.26 | 0.06 | 0.31 | | 4.44 | 0.32 |
| Mid temporal | MEM | 0.10 | 0.05 | **0.42** | **<0.001** | **0.22** | **<0.001** | **0.17** | **<0.001** | | 19.36 | 0.06 |
|  | EF | 0.10 | 0.05 | **0.38** | **<0.001** | 0.10 | 0.08 | 0.06 | 0.26 | | 34.36 | 0.06 |
|  | LAN | 0.10 | 0.05 | **0.36** | **<0.001** | 0.10 | 0.08 | 0.06 | 0.26 | | 35.72 | 0.05 |
|  | VS | 0.10 | 0.05 | **0.19** | **0.01** | 0.07 | 0.26 | 0.05 | 0.42 | | 19.04 | 0.07 |
| Neurogranin | MEM | -0.07 | 0.41 | -0.05 | 0.60 | 0.06 | 0.52 | 0.06 | 0.54 | | 0.27 | 0.81 |
|  | EF | -0.07 | 0.41 | 0.04 | 0.67 | -0.13 | 0.13 | -0.13 | 0.14 | | 0.51 | 0.84 |
|  | LAN | -0.07 | 0.41 | -0.07 | 0.40 | 0.05 | 0.57 | 0.04 | 0.62 | | 1.45 | 0.65 |
|  | VS | -0.07 | 0.41 | 0.01 | 0.99 | -0.05 | 0.58 | -0.05 | 0.58 | | 0.09 | 0.99 |
| sTREM2 | MEM | -0.08 | 0.19 | -0.10 | 0.07 | -0.10 | 0.07 | **0.19** | **0.01** | | 2.64 | 0.28 |
|  | EF | -0.08 | 0.19 | -0.08 | 0.15 | **0.12** | **0.04** | 0.11 | 0.05 | | 3.44 | 0.32 |
|  | LAN | -0.08 | 0.19 | -0.06 | 0.23 | **0.14** | **0.01** | **0.14** | **0.02** | | 2.34 | 0.44 |
|  | VS | -0.08 | 0.19 | -0.05 | 0.36 | 0.06 | 0.34 | 0.05 | 0.38 | | 2.67 | 0.51 |
| YKL-40 | MEM | -0.04 | 0.78 | -0.06 | 0.68 | 0.10 | 0.51 | 0.09 | 0.52 | | 0.43 | 0.99 |
|  | EF | -0.04 | 0.78 | -0.13 | 0.34 | -0.11 | 0.43 | -0.12 | 0.41 | | 0.24 | 0.82 |
|  | LAN | -0.04 | 0.78 | **-0.31** | **0.03** | 0.26 | 0.07 | 0.25 | 0.07 | | 2.58 | 0.84 |
|  | VS | -0.04 | 0.78 | -0.06 | 0.66 | 0.01 | 0.98 | 0.01 | 0.99 | | 0.05 | 0.99 |
| **Longitudinal** |  |  |  |  |  |  |  |  |  | |  |  |
| p-tau | MEM | **-0.18** | **0.01** | **-0.25** | **<0.001** | **0.22** | **<0.001** | **0.19** | **0.01** | | **16.82** | **0.01** |
|  | EF | **-0.18** | **0.01** | -0.09 | 0.14 | **0.21** | **0.01** | **0.20** | **0.01** | | 4.65 | 0.33 |
|  | LAN | **-0.18** | **0.01** | **-0.18** | **0.01** | **0.16** | **0.02** | **0.14** | **0.05** | | **16.08** | **0.02** |
|  | VS | **-0.18** | **0.01** | -0.01 | 0.87 | **0.16** | **0.02** | **0.16** | **0.02** | | 1.05 | 0.84 |
| t-tau | MEM | **-0.14** | **0.04** | **-0.22** | **<0.001** | **0.22** | **<0.001** | **0.20** | **0.01** | | 12.05 | 0.05 |
|  | EF | **-0.14** | **0.04** | **-0.20** | **0.01** | **0.21** | **0.01** | **0.19** | **0.01** | | **10.21** | **0.04** |
|  | LAN | **-0.14** | **0.04** | **-0.16** | **0.01** | **0.16** | **0.02** | **0.14** | **0.03** | | 10.74 | 0.07 |
|  | VS | **-0.14** | **0.04** | -0.07 | 0.30 | **0.16** | **0.02** | **0.15** | **0.03** | | 2.65 | 0.50 |
| NFL | MEM | 0.11 | 0.16 | -0.07 | 0.42 | **0.25** | **0.01** | **0.26** | **0.01** | | 3.86 | 0.38 |
|  | EF | 0.11 | 0.16 | -0.14 | 0.11 | **0.24** | **0.01** | **026** | **0.01** | | 6.94 | 0.17 |
|  | LAN | 0.11 | 0.16 | -0.09 | 0.30 | 0.17 | 0.05 | **0.19** | **0.04** | | 5.04 | 0.34 |
|  | VS | 0.11 | 0.16 | -0.16 | 0.07 | 0.13 | 0.13 | 0.15 | 0.08 | | 12.11 | 0.17 |
| Whole brain | MEM | 0.05 | 0.56 | **0.32** | **<0.001** | **0.22** | **0.01** | **0.21** | **0.01** | | 6.13 | 0.54 |
|  | EF | 0.05 | 0.56 | **0.28** | **<0.001** | **0.20** | **0.01** | **0.19** | **0.01** | | 5.61 | 0.60 |
|  | LAN | 0.05 | 0.56 | **0.26** | **<0.001** | 0.15 | 0.06 | 0.14 | 0.06 | | 7.63 | 0.59 |
|  | VS | 0.05 | 0.56 | **0.24** | **0.01** | 0.13 | 0.10 | 0.12 | 0.13 | | 6.93 | 0.56 |
| Hippocampus | MEM | 0.13 | 0.08 | **0.50** | **<0.001** | **0.22** | **0.01** | **0.16** | **0.02** | | 27.06 | 0.07 |
|  | EF | 0.13 | 0.08 | **0.42** | **<0.001** | **0.21** | **0.01** | **0.16** | **0.03** | | 24.22 | 0.08 |
|  | LAN | 0.13 | 0.08 | **0.43** | **<0.001** | 0.15 | 0.06 | 0.10 | 0.16 | | 32.94 | 0.07 |
|  | VS | 0.13 | 0.08 | **0.28** | **<0.001** | 0.13 | 0.10 | 0.09 | 0.22 | | 23.47 | 0.07 |
| Entorhinal | MEM | **0.21** | **0.01** | **0.41** | **<0.001** | **0.22** | **0.01** | **0.14** | **0.05** | | **36.07** | **0.01** |
|  | EF | **0.21** | **0.01** | **0.35** | **<0.001** | **0.20** | **0.01** | 0.14 | 0.06 | | **32.78** | **0.01** |
|  | LAN | **0.21** | **0.01** | **0.43** | **<0.001** | 0.15 | 0.06 | 0.07 | 0.35 | | 55.45 | <0.001 |
|  | VS | **0.21** | **0.01** | **0.21** | **0.01** | 0.13 | 0.10 | 0.09 | 0.26 | | 26.40 | 0.03 |
| Mid temporal | MEM | 0.14 | 0.08 | **0.53** | **<0.001** | **0.22** | **0.01** | **0.15** | **0.04** | | 32.13 | 0.09 |
|  | EF | 0.14 | 0.08 | **0.47** | **<0.001** | **0.20** | **0.01** | **0.16** | **0.04** | | 29.65 | 0.07 |
|  | LAN | 0.14 | 0.08 | **0.55** | **<0.001** | 0.15 | 0.06 | 0.08 | 0.23 | | 45.89 | 0.07 |
|  | VS | 0.14 | 0.08 | **0.34** | **<0.001** | 0.13 | 0.10 | 0.08 | 0.27 | | 31.47 | 0.04 |
| sTREM2 | MEM | -0.09 | 0.29 | -0.10 | 0.24 | **0.23** | **0.01** | **0.22** | **0.01** | | 2.00 | 0.47 |
|  | EF | -0.09 | 0.29 | -0.02 | 0.78 | **0.26** | **0.01** | **0.26** | **0.01** | | 0.08 | 0.94 |
|  | LAN | -0.09 | 0.29 | -0.09 | 0.31 | **0.19** | **0.03** | **0.19** | **0.04** | | 1.96 | 0.58 |
|  | VS | -0.09 | 0.29 | 0.03 | 0.73 | 0.18 | 0.05 | **0.18** | **0.04** | | 1.96 | 0.58 |
| YKL-40 | MEM | 0.18 | 0.19 | 0.07 | 0.62 | 0.24 | 0.08 | 0.23 | 0.09 | | 1.62 | 0.79 |
|  | EF | 0.18 | 0.19 | -0.06 | 0.63 | 0.11 | 0.43 | 0.12 | 0.37 | | 2.46 | 0.63 |
|  | LAN | 0.18 | 0.19 | -0.02 | 0.90 | 0.11 | 0.44 | 0.11 | 0.42 | | 1.67 | 0.80 |
|  | VS | 0.18 | 0.19 | 0.02 | 0.90 | 0.02 | 0.85 | 0.02 | 0.86 | | 0.10 | 0.97 |

Significant effects (P <0.05) are shown in bold. Models included age, sex, education, *APOEε4* status and intracranial volume as covariates.

Abbreviations: MCI mild cognitive impairment; *APOEε4*, Apolipoprotein E4; p-tau, Phosphorylated tau; t-tau, Total tau; NFL, Neurofilament light; sTREM2, Soluble triggering receptor on myeloid cells 2; MEM, Memory function; EF, Executive function; LAN, Language; VS, Visuospatial functioning.
